# Red Clover HDT, a BAHD Hydroxycinnamoyl-Coenzyme A:L-3,4-Dihydroxyphenylalanine (L-DOPA) Hydroxycinnamoyl Transferase That Synthesizes Clovamide and Other *N*-Hydroxycinnamoyl-Aromatic Amino Acid Amides

**DOI:** 10.3389/fpls.2021.727461

**Published:** 2021-11-09

**Authors:** Michael L. Sullivan, Benjamin J. Knollenberg

**Affiliations:** ^1^US Dairy Forage Research Center, USDA-ARS, Madison, WI, United States; ^2^Department of Plant Sciences, Pennsylvania State University, University Park, PA, United States

**Keywords:** BAHD hydroxycinnamoyl-CoA hydroxycinnamoyl transferase, clovamide, CYP76AD6, hydroxycinnamoyl amide, phenylpropanoid, red clover, tyrosine hydroxylase

## Abstract

Red clover leaves accumulate high levels (up to 1 to 2% of dry matter) of two caffeic acid derivatives: phaselic acid (2-*O*-caffeoyl-L-malate) and clovamide [*N*-caffeoyl-L-3,4-dihydroxyphenylalanine (L-DOPA)]. These likely play roles in protecting the plant from biotic and abiotic stresses but can also help preserve protein during harvest and storage of the forage *via* oxidation by an endogenous polyphenol oxidase. We previously identified and characterized, a hydroxycinnamoyl-coenzyme A (CoA):malate hydroxycinnamoyl transferase (HMT) from red clover. Here, we identified a hydroxycinnamoyl-CoA:L-DOPA hydroxycinnamoyl transferase (HDT) activity in unexpanded red clover leaves. Silencing of the previously cloned HMT gene reduced both HMT and HDT activities in red clover, even though the HMT enzyme lacks HDT activity. A combination of PCR with degenerate primers based on BAHD hydroxycinnamoyl-CoA transferase sequences and 5′ and 3′ rapid amplification of cDNA ends was used to clone two nearly identical cDNAs from red clover. When expressed in *Escherichia coli*, the encoded proteins were capable of transferring hydroxycinnamic acids (*p*-coumaric, caffeic, or ferulic) from the corresponding CoA thioesters to the aromatic amino acids L-Phe, L-Tyr, L-DOPA, or L-Trp. Kinetic parameters for these substrates were determined. Stable expression of HDT in transgenic alfalfa resulted in foliar accumulation of *p*-coumaroyl- and feruloyl-L-Tyr that are not normally present in alfalfa, but not derivatives containing caffeoyl or L-DOPA moieties. Transient expression of HDT in *Nicotiana benthamiana* resulted in the production of caffeoyl-L-Tyr, but not clovamide. Coexpression of HDT with a tyrosine hydroxylase resulted in clovamide accumulation, indicating the host species’ pool of available amino acid (and hydroxycinnamoyl-CoA) substrates likely plays a major role in determining HDT product accumulation in planta. Finally, that HDT and HMT proteins share a high degree of identity (72%), but differ substantially in substrate specificity, is promising for further investigation of structure-function relationships of this class of enzymes, which could allow the rational design of BAHD enzymes with specific and desirable activities.

## Introduction

**Figure 1:**
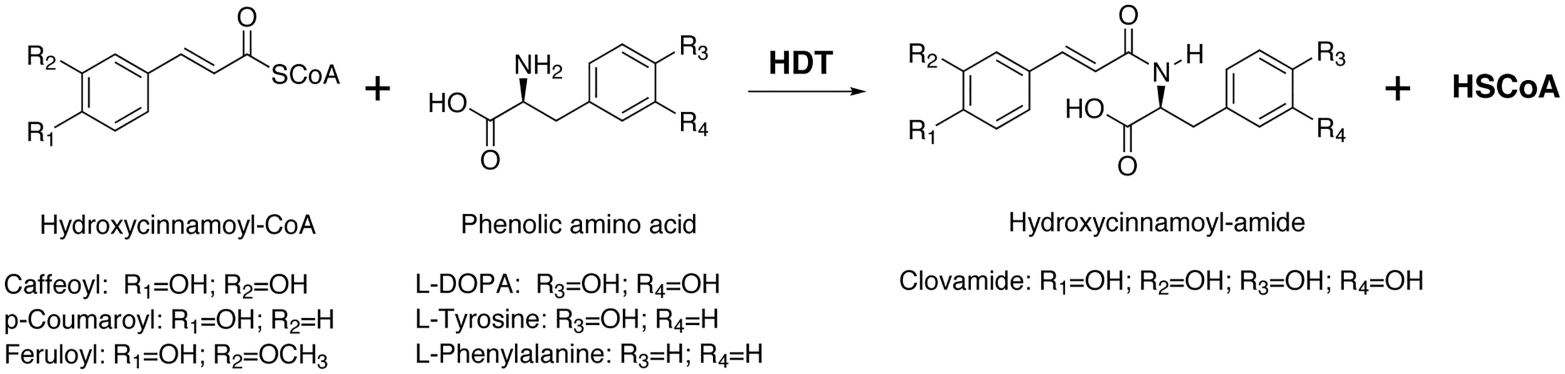
Formation of clovamide and related hydroxycinnamoyl amides by a hydroxycinnamoyl-CoA:L-DOPA hydroxycinnamoyl transferase, HDT. The enzyme transfers a hydroxycinnamoyl moiety from CoA to the amine group on a phenolic amino acid acceptor to form the corresponding hydroxycinnamoyl amide and free CoA (HSCoA). L-Trp and L-Leu can also serve as acceptors for amide formation (not shown).

Clovamide (CAS 53755-02-5), an amide of caffeic acid with 3,4-dihydroxy-L-phenylalanine (L-DOPA; Figure 1), was first identified from leaves and stems of red clover (*Trifolium pratense* L.; Yoshihara et al., 1974). Clovamide and related hydroxycinnamoyl amides have subsequently been identified in other plants, including *Dalbergia melanoxylon* Guill. & Perr (African blackwood; Van Heerden et al., 1980) and *Theobroma cacao* L. (cocoa tree; Sanbongi et al., 1998). There has been substantial interest in clovamide and related compounds for their potential to be antioxidants and their potential for other bioactive/pharmacological properties (see, for example, Zeng et al., 2011; Kolodziejczyk-Czepas et al., 2017). Despite the interest in clovamide as a bioactive compound, little work has been carried out to elucidate how it is made in planta.

In red clover, *trans*-clovamide can accumulate to relatively high levels: up to around 1% of dry matter in fully expanded mature leaves (Winters et al., 2008; Sullivan and Zeller, 2013). There are some indications that levels of clovamide are influenced by genotype (Sullivan and Zeller, 2013) and also by environment (Winters et al., 2008). In planta, high levels of phenolic compounds like clovamide might play a role in protecting plants from biotic (e.g., pathogenesis and herbivory) and abiotic stresses (e.g., UV radiation and ozone). Consistent with a role in protecting the plant from biotic stresses is the observation that clovamide accumulates in red clover roots in response to jasmonic acid treatment (Tebayashi et al., 2000). Also, in red clover, clovamide, along with 2-*O*-caffeoyl-L-malate (phaselic acid), is a substrate for an endogenous polyphenol oxidase (PPO). In the case of clovamide, presumably both the caffeoyl moiety and the L-DOPA moiety are subject to oxidation by PPO. Upon tissue breakdown and release of PPO from plastids, oxidation of *o*-diphenol moieties, such as caffeic acid or L-DOPA (with such phenolics presumably present in the cytosol or vacuole), to the corresponding reactive quinones and the subsequent secondary reaction of those quinones leads to the familiar post-harvest browning reaction seen with produce and other plant materials (e.g., red clover hay; Steffens et al., 1994; Kagan et al., 2016). There is evidence that PPO-mediated quinone formation helps protect plants from biotic stress (Thipyapong et al., 2004). Recently, in *Theobroma cacao*, clovamide (and presumably related hydroxycinnamoyl amides) has been shown to be a resistance factor against *Phytophthora* species, with the possibility of both PPO-dependent and -independent mechanisms involved (Knollenberg et al., 2020). Additionally, we and others have shown that PPO-mediated oxidation of caffeic acid derivatives and other *o*-diphenols by PPO protects forage protein from post-harvest degradation (Sullivan and Hatfield, 2006; Winters et al., 2008; Sullivan and Zeller, 2013). This natural system of protein protection is present in red clover and perennial peanut (Sullivan and Foster, 2013), but absent in many important forages, such as alfalfa, which fails both to express PPO and accumulate PPO substrates in its leaves. Because degraded protein is poorly utilized by ruminant animals, transferring this natural system of protein protection to forages, such as alfalfa or corn (as silage), could save US farmers $2 billion annually and prevent release of nitrogen into the environment (as a result of poor protein utilization).

For this reason, we have been trying to understand how red clover makes and accumulates high levels of PPO-oxidizable phaselic acid and clovamide. Although in the Brassicaceae, hydroxycinnamoyl-malate esters are synthesized *via* a hydroxycinnamoyl-glucose transferase (Lehfeldt et al., 2000), in red clover, we identified a BAHD family (D'Auria, 2006) hydroxycinnamoyl-coenzyme A (CoA):malate hydroxycinnamoyl transferase (originally called HCT2, now HMT) crucial for phaselic acid biosynthesis (Sullivan, 2009; Sullivan and Zarnowski, 2011). Similar BAHD family hydroxycinnamoyl-CoA transferases have been implicated in the biosynthesis of *p*-coumaroyl-shikimate (Hoffmann et al., 2003), chlorogenic acid (Niggeweg et al., 2004; Lepelley et al., 2007), rosmarinic acid (Berger et al., 2006), avenanthramides (Wise et al., 2009), and other hydroxycinnamic acid derivatives (Sullivan, 2017). Here, we describe the identification of a BAHD hydroxycinnamoyl-CoA:L-DOPA hydroxycinnamoyl transferase (HDT) capable of making clovamide and related hydroxycinnamoyl-aromatic amino acid amides *via* the reaction shown in Figure 1.

## Materials and Methods

### Reagents

Purchased reagents were of molecular biology grade or higher. *trans*-Clovamide and *trans*-caffeoyl-L-tyrosine, for use as standards, were purchased from Santa Cruz Biotechnology (Dallas, TX, United States) and rosmarinic acid was purchased from Cayman Chemical (Ann Arbor, MI, United States). *trans*-*p*-Coumaroyl-, -caffeoyl-, and -feruloyl-CoA thioesters were prepared using recombinant *Arabidopsis thaliana* (L.) Hynth. 4CL1 protein (Lee et al., 1995) expressed in *Escherichia coli* using the pET30 expression vector (MilliporeSigma, St. Louis, MO, United States) and quantified as previously detailed (Sullivan, 2009).

### Plant Material

For experiments with red clover (*Trifolium pratense* L.), a highly regenerable genotype (designated NRC7) derived from a population of NewRC germplasm (Smith and Quesenberry, 1995) was used. For transgenic alfalfa, a highly regenerable clone of Regen-SY (Bingham, 1991) was used. A collection of NRC7 red clover plants silenced for red clover hydroxycinnamoyl-CoA:malate hydroxycinnamoyl transferase (HMT) was generated as described by Sullivan and Zarnowski (2011) using the RNAi construct described therein which contains a hairpin RNA corresponding to the region between nucleotides 481 and 1,224 of GenBank sequence EU861219. *Nicotiana benthamiana* Domin plants, grown from seeds (4–5weeks old), were used for transient expression experiments.

### Extraction of Phenolic Compounds From Plant Tissues

For all tissues except *N. benthamiana*, tissue samples were ground in liquid nitrogen in a mortar and pestle, or for small samples in a 2ml screw cap tube with two 4mm glass beads using a Mini-Beadbeater (Biospec Products, Bartlesville, OK, United States). The ground frozen tissue was extracted at room temperature with 10ml/g fresh weight (FW) 100mM HCl, 50mM ascorbic acid for 30min with periodic mixing. Extracts were filtered through Miracloth (MilliporeSigma) or glass wool and then centrifuged at 20,000×g at room temperature. 1ml of the resulting supernatant was applied to a 1ml ENVI-18 solid phase extraction column (MilliporeSigma) preequilibrated with 3×1ml methanol and 3×1ml 0.1% (v/v) acetic acid in water, pH adjusted to 2.5 with HCl. The column was washed with 3×1ml 0.1% acetic acid (v/v) in water (pH 2.5 with HCl) and eluted with 1ml methanol.

For *N. benthamiana* leaf tissue, two leaf disks (15mm diameter) from two leaves on the same plant were punched out with a cork borer and placed in a 2ml screw cap tube containing 1ml of 80/20/0.1 methanol/water/formic acid (v/v/v). Tubes were heated to 80°C for 30min. Supernatants were removed to a fresh tube, evaporated to dryness in a SpeedVac, and the resulting pellets were re-dissolved in an equal volume of 10/90/0.1 methanol/water/formic acid (v/v/v) with 2.5μg/ml rosmarinic acid as an internal standard. Samples were filtered through a 0.2μm nylon spin filter column (Norgen Biotek Corp., Thorold, Canada) prior to analysis.

### HPLC and Mass Spectrometry

With the exception of those from *N. benthamiana* leaf tissue (see below), phenolic samples (from red clover or alfalfa leaves or from *in vitro* reactions) were analyzed by HPLC on a Shim-Pack XR-ODS II (C-18) 120Å column (Shimadzu Scientific Instruments North America, Columbia, MD, United States; 100×2.0mm×2.2 micron) using a two solvent system [Solvent A: deionized water with 0.1% (v/v) formic acid; Solvent B: acetonitrile] as previously described (Sullivan, 2014). Compound elution was monitored (250 to 500nm) with a UV/visible photodiode array detector (PDA). In most cases, elution was also monitored with a MS2020 mass spectrometer (MS; Shimadzu Scientific Instruments North America) using a dual-ion source (electrospray and atmospheric pressure chemical ionization) with data collection in both positive and negative ion modes. MS data were collected between 2.0 and 17.0min of the HPLC run, scanning for (*m/z*) between 50 and 500 at 7500 u/s, with detector voltage of 1.3kV, nebulizing gas flow of 1.5l/min, drying gas flow of 10l/min, and desolvation line and heat block temperatures of 250°C.

Compounds of interest were quantified from peak areas of PDA chromatograms (250–500nm) using HPLC Solutions software (Shimadzu Scientific Instruments North America) and a standard curve generated using 20, 10, and 5mg/ml of purchased clovamide (for quantitation of clovamide or other hydroxycinnamoyl amides) or free hydroxycinnamic acids for hydroxycinnamoyl esters with shikimate or malate as detailed previously (Sullivan, 2009).

Extracts from *N. benthamiana* leaf tissue were analyzed on an Agilent 1260 Infinity HPLC system (Agilent Technologies, Santa Clara, CA, United States) equipped with a fluorescence detector (FLD, Agilent 1260) and an Agilent Poroshell EC-C18 column (150×3mm, 2.7μm) with an aqueous methanol gradient as described by Knollenberg et al. (2020). Clovamide and caffeoyl-L-Tyr were monitored and quantitated using a FLD with excitation at 320nm and emission at 440nm and photomultiplier tube (PMT) gain set to 16. Purchased clovamide and caffeoyl-L-Tyr were measured at 16, 4, 1, and 0.2μg/ml to generate calibration curves (*r^2^*=0.9998 and 1, respectively).

### Preparation of Plant Protein Extracts for Enzyme Activity

Tissue was powdered in liquid nitrogen using a mortar and pestle or Mini-Beadbeater as described above and extracts in 100mM sodium phosphate buffer were prepared and low molecular weight compounds removed by gel filtration spin columns essentially as described previously (Sullivan, 2014). Extracts were divided into 150 to 200μl aliquots, flash frozen in liquid nitrogen, and stored at −80°C until needed. In the case of pH adjustment by the spin column procedure, pH was confirmed by spotting a small amount of extract on pH indicator paper. Protein content of the extracts was determined using Bio-Rad Protein Assay (Bio-Rad Laboratories, Hercules, CA, United States) using bovine serum albumin as the standard.

### Preparation of RNA and cDNA, Plasmid Preparation, and Sequence Analysis

Total RNA was prepared from plant tissues using the RNeasy Plant Mini Kit (Qiagen, Germantown, MD, United States). Oligo dT-primed cDNA was prepared using Superscript III reverse transcriptase according to the manufacturer’s protocol (Invitrogen, Carlsbad, CA, United States) from DNase I-treated total RNA. Plasmid DNA was prepared using the QIAprep Spin Miniprep Kit (Qiagen). DNA sequence was determined by Sanger cycle sequencing using Big Dye v3.1 (Applied Biosystems, Foster City, CA, United States) and run on ABI 3730xl DNA Analyzers by the University of Wisconsin Biotechnology Center (Madison, WI, United States). Sequence analyses were carried out using Lasergene Version 12 or higher (DNAStar, Madison, WI, United States) and BLAST programs using the National Center for Biotechnology Information (NCBI,^1^) web site. Phylogenetic analysis was carried out using http://www.phylogeny.fr/index.cgi (Dereeper et al., 2008), an online tool. The workflow included sequence alignment by MUSCLE without the optional curation by Gblocks, phylogeny by PhyML with branch confidence determined by the approach of Anisimova and Gascuel (2006) and tree rendering by TreeDyn. Besides red clover HDT1 and HDT2, analyzed protein sequences included representative *Medicago truncatula* sequences from Clade Vb of the eight clades defined by Tuominen et al. (2011), as well as several Clade Vb BAHD hydroxycinnamoyl-CoA transferases that have been biochemically characterized. GenBank identifiers for the analyzed protein sequences are provided in the figure.

### Cloning of HDT by PCR

Degenerate oligonucleotide primers based on conserved regions of previously cloned red clover HMT and hydroxycinnamoyl-CoA:shikimate hydroxycinnamoyl transferase (HST; GenBank accessions EU861219 and EU861218, respectively) and two sequences from *Phaseolus vulgaris* L.; hydroxycinnamoyl-CoA:tetrahydroxyhexanedioic acid hydroxycinnamoyl transferase (HHHT; Sullivan, 2017) and a second uncharacterized, but likely hydroxycinnamoyl transferase (GenBank Accessions KX443573 and XM_007146336, respectively); were used in a nested PCR approach to obtain a DNA fragment corresponding to HDT (see also Supplementary Figure S1 which details primer annealing sites on the HDT sequence). First-round PCR reactions (50μl) contained 1×Phusion HF Buffer, 200μM dNTP, cDNA equivalent to 0.1μg total RNA from unexpanded red clover leaves (prepared as described above), 1 unit Phusion DNA polymerase (New England Biolabs, Ipswich, MA, United States), 1μM each primers ms809 and ms815 (Table 1). The PCR reaction was incubated for 30s at 98°C in the preheated block of a thermocycler. This was followed by 35cycles of 98°C for 10s (denaturation), 55°C for 20s (annealing), and 72°C for 30s (extension) followed by a final 5min extension at 72°C. 20μl of the PCR reaction was resolved *via* electrophoresis on a 1.0% agarose gel using standard techniques. An approximately 1,000bp DNA fragment was excised from the gel and purified using QiaEx Resin (Qiagen) according to the manufacturer’s procedure.

**Table 1:** Oligo nucleotide primers used in this study.

Designation	Sequence^a^ (5′ to 3′)
ms268	GTGTGAGTCACACTGTGCCAATC
ms269	ACGGCCAGCAAGATCCAA
ms701	GGCATTAACTGCAACAAAAGGATGTG
ms702	CCAAGAAACAATTTCATTGCATCATCCATC
ms809	TWYTAYCCWDTRGCTGGHMG
ms815	AYWGSCYTYCCMYAWCCAAAATC
ms867	AMTTCATCAAYWCATGGKC
ms870	CCAAAATCWGMWTCRTRAAHVGG
ms881	CTGGATACCTAGAACATCTTCTTCATTGGC
ms882	CACCTTTGGAGCCACGTTTTGAACACTTGG
ms884	CAACACAGAACTTCAASCTAGCATACC
ms885	ACCAACTTAGAGGGTGATTTTGGGTC
ms886	CGGGCCATGGTAACCATTATAGCTTCTCAC
ms887	GGGCCTCGAGTCATATCTCCTCATAAAAATACTTGTT
ms888	GTCTAGA*AAACA*ATGGTAACCATTATAGCTTCTCAC
ms889	CGGTACCTCATATCTCCTCATAAAAATACTTGTT
bk1	CCTAGGATGGTAACCATTATAGCTTCTCACAC
bk2	GTTAACTCATATCTCCTCATAAAAATACTTGTTGA
ms1008	ACTTTGCTCCCACACTTGAG
ms1009	TGTTGCATACCAACACCAAG
ms1010	CATAGAAGGTGGTAGATTAGAATTGA
ms1011	TGGAACAAGTTCTTTGATGGTG

a Where appropriate, introduced restriction sites are underlined and an introduced dicot translational consensus sequence (Joshi et al., 1997) is italicized.

Second round (nested) PCR reactions (25μl) contained 1×Phusion HF Buffer, 200μM dNTP, gel purified first-round PCR product equivalent to 0.01μl of the first-round reaction, 0.5units Phusion DNA polymerase, 0.5μM each primers ms867 and ms870 (Table 1). The PCR reaction was incubated for 30s at 98°C in the preheated block of a thermocycler. This was followed by 35cycles of 98°C for 10s (denaturation), 49°C for 20s (annealing), and 72°C for 30s (extension) followed by a final 5min extension at 72°C. 20μl of the PCR reaction was resolved *via* electrophoresis on a 1.0% agarose gel using standard techniques. An approximately 700bp DNA fragment was excised from the gel and purified using QiaEx Resin according to the manufacturer’s procedure. The DNA fragment was ligated into pGEM T-Easy (Promega Corporation, Madison, WI, United States) according to the manufacturer’s protocol.

Sequence of the resulting DNA fragment was used to design primers for 5′ and 3′ rapid amplification of cDNA ends (RACE). 5′ and 3′ RACE were carried out using the SMARTer RACE cDNA Amplification Kit (Catalog # 634923, Clontech Laboratories, Mountain View, CA, United States) using total RNA from unexpanded red clover leaves and ms881 and ms882 (Table 1) as the gene specific primers for 5′ and 3′ RACE, respectively. The resulting 5′ and 3′ RACE products were gel purified and ligated into pGEM T-Easy as described above. DNA sequence of the resulting fragments was used to design primers ms884 and ms885 (Table 1) for end-to-end PCR. Three independent end-to-end PCR reactions (25μl each) contained 1×Phusion HF Buffer, 200μM dNTP, first strand 5′ RACE cDNA (equivalent to 0.017μg total RNA), 0.5units Phusion DNA polymerase, 0.5μM each primers ms884 and ms885. The PCR reactions were incubated for 30s at 98°C in the preheated block of a thermocycler. This was followed by 30cycles of 98°C for 10s (denaturation), 69°C for 20s (annealing), and 72°C for 1min (extension) followed by a final 5min extension at 72°C. The resulting DNA fragments were gel purified and ligated into pGEM T-Easy (Promega Corporation) as described above and four clones from each of the three independent PCR reactions were sequenced.

### Evaluation of HDT mRNA Levels by Quantitative Real-Time PCR

To assess mRNA levels of HDT, HST, and HMT, cDNA was prepared from DNase I-treated total RNA isolated from unexpanded and mature red clover leaves as described above. Quantitative real-time PCR was carried out using PowerUp SYBR Green PCR Master Mix (Thermo Fisher Scientific, Waltham, MA, United States) in triplicate 10μl reactions. Each reaction contained cDNA equivalent to 12.5ng total RNA and primers at 300nM. Primer pairs (Table 1) were ms1010/ms1011 to detect HDT1/2, ms1008/ms1009 to detect HST (HCT1A/B, GenBank EU861218, FJ151489), ms701/ms702 to detect HMT (GenBank EU861219), and ms268/ms269 to detect actin (GenBank AY372368) as the reference gene. Real-time PCR was run in a QuantStudio 5 (Thermo Fisher Scientific) using the cycling conditions 50°C for 2min, 95°C for 2min, followed by 40cycles of 95°C for 15s, 58°C for 15s, and 72°C for 1min with SYBR as the reporter and ROX as the passive reference. Following cycling melt curves were generated using the default settings (65°C to 95°C over 400s). Threshold cycle (CT) was determined using the auto function of the QuantStudio analysis software.

### Plasmids for Red Clover HDT Heterologous Expression Experiments

For all gene constructions, standard molecular biology techniques were used (Sambrook et al., 1989; Ausubel et al., 1998). When fragments for cloning were generated *via* PCR, the cloned insert was sequenced to ensure no mutations were introduced that would alter the sequence of the translated protein.

For initial studies of the enzymatic properties of the protein, plasmids containing full-length red clover HDT coding regions were used as templates in PCR reactions with primers (Table 1) designed to introduce an NcoI restriction site at the start codons (ms886) and an XhoI site immediately following the stop codon (ms887) of each open reading frame using Phusion polymerase with reaction and cycling conditions recommended by the manufacturer. The resulting PCR products were digested with NcoI and XhoI and inserted into pET28a (MilliporeSigma) digested with NcoI and XhoI. To produce protein for kinetic analyses, an HDT1 open reading frame (GenBank Accession MF536893), codon-optimized for expression in *E. coli*, was provided by Lytic Solutions (Madison, WI, United States) and ligated into expression vector pET28 (MilliporeSigma) such that a histidine tag sequence (MGSSHHHHHHSSGLVPRGSH) was fused to the N-terminus of the HDT protein as described elsewhere (Sullivan and Bonawitz, 2018).

For stable expression in transgenic alfalfa, PCR primer pairs (Table 1) were designed to introduce XbaI (ms888) and KpnI (ms889) restriction endonuclease sites flanking the 5′ and 3′ ends of the coding regions of the two red clover HDT genes. Additionally, the forward primer provided the proposed dicot consensus sequence AAACA (Joshi et al., 1997) immediately upstream of the initiating methionine codon. This primer pair was used in PCR reactions with plasmids containing the full-length red clover HDT coding regions as templates using Phusion polymerase with reaction and cycling conditions recommended by the manufacturer. The resulting PCR fragments were ligated as XbaI-KpnI fragments downstream of the CsVMV promoter (Verdaguer et al., 1996) in pMLS312 (Verdonk and Sullivan, 2013), a derivative of pBIB-HYG plant transformation vector (Becker, 1990).

For transient expression in *N. benthamiana*, the HDT1 coding sequence (GenBank MF115997.1) was amplified by PCR with Phusion polymerase using primers bk1 and bk2 (Table 1), which introduced AvrII and HpaI restriction enzyme sites at the 5′ and 3′ ends, respectively. The resulting fragment was ligated into pMiniT 2.0 (New England Biolabs). The coding sequence was subcloned from pMiniT 2.0 as an AvrII and HpaI fragment ligated into pGZ12.0501 (GenBank KF871320.1) digested with SpeI and HpaI, putting the coding sequence after the E12-Ω CaMV-35S constitutive promoter (Mitsuhara et al., 1996). pGZ12.0501 is derived from pGH00.0126 (GenBank KF018690.1; Maximova et al., 2003), which lacks this over-expression cassette. pGH00.0126 served as the empty vector control.

For transient expression in *N. benthamiana* of beet (*Beta vulgaris* L.) CYP76AD6, a tyrosine hydroxylase that converts L-Tyr into L-DOPA (Polturak et al., 2016), the construct “3alpha2-35S-CYP76AD6-tAct2” was kindly provided by Asaph Aharoni (Weizmann Institute of Science). This construct contains the coding sequence of *B. vulgaris* CYP76AD6 expressed from the CaMV35S promoter. The plasmid “pDGB3alpha2_35S:P19:Tnos” for over-expression of the p19 silencing suppressor in transient expression experiments was purchased from Addgene (Watertown, MA, United States; plasmid #GB1203; Sarrion-Perdigones et al., 2013).

### *In vitro* Hydroxycinnamoyl-CoA Transferase Activity Assays

General *in vitro* reactions for hydroxycinnamoyl-CoA transferase activity contained 100mM sodium phosphate buffer (pH 7.5), 25 to 50mM ascorbate, 1 to 2mM *p*-coumaroyl-, caffeoyl-, or feruloyl-CoA donor substrate, 1 to 6mM acceptor substrate (L-amino acids, shikimic acid, or malic acid), and red clover leaf or *E. coli* extract. When red clover extract was the enzyme source, reaction head space was exchanged for nitrogen to prevent oxidation of caffeoyl and L-DOPA moieties by endogenous PPO. Reactions were incubated at 30°C for up to 1h prior to being stopped by the addition of one-fifth volume of 10% formic acid. Precipitated protein was removed by centrifugation (17,000×g for 5min at room temperature) and the supernatant analyzed for reaction products by HPLC as described above.

### Determination of Kinetic Parameters of HDT

Kinetic parameters for HDT were determined by measuring initial reaction rates by release of free CoA in real time using DTNB [5,5′-dithio-*bis*-(2-nitrobenzoic acid)] and spectroscopy as detailed elsewhere (Sullivan and Bonawitz, 2018). Measurements were made in a temperature-controlled spectrophotometer at 25°C. Reactions were carried out in 100mM sodium phosphate, 1mM EDTA, pH 8.0. Acceptor substrates were prepared in 1N HCl as 66.7×stock solutions, so an equal amount of 1N NaOH was added to reactions to neutralize the HCl (15mM final). Substrate and enzyme amounts used are detailed in Table 2.

**Table 2:** Reaction conditions for determination of kinetic parameters.

Variable substrate	Range (μM)	Number of condition	Constant substrate	HDT1 protein (μg/ml)
*p*-Coumaroyl-CoA	1 to 50	10	L-Tyr	0.44
*p*-Coumaroyl-CoA	1 to 50	10	L-DOPA	0.44
*p*-Coumaroyl-CoA	0.5 to 75	11	L-Trp	1.00
Caffeoyl-CoA	1.25 to 150	10	L-Tyr	0.20
Caffeoyl-CoA	1.25 to 150	10	L-DOPA	0.23
Caffeoyl-CoA	0.5 to 75	10	L-Trp	0.50
Feruloyl-CoA	1 to 50	10	L-Tyr	1.64
Feruloyl-CoA	1 to 50	10	L-DOPA	1.09
Feruloyl-CoA	0.5 to 75	11	L-Trp	2.00
L-Tyr	50 to 3,000	10	*p*-Coumaroyl-CoA	0.44
L-Tyr	100 to 3,000	8	Caffeoyl-CoA	0.20
L-Tyr	7.5 to 3,000	15	Feruloyl-CoA	1.64
L-DOPA	100 to 10,000	11	*p*-Coumaroyl-CoA	0.44
L-DOPA	250 to 10,000	9	Caffeoyl-CoA	0.20
L-DOPA	50 to 10,000	13	Feruloyl-CoA	1.64
L-Trp	100 to 15,000	10	*p*-Coumaroyl-CoA	2.00
L-Trp	100 to 15,000	10	Caffeoyl-CoA	2.00
L-Trp	50 to 10,000	11	Feruloyl-CoA	2.00

All kinetic data analyses were carried out using GraphPad Prism version 7 or higher for Mac OS (GraphPad Software, La Jolla, CA, United States). Each analysis (i.e., series of substrate concentrations) was carried out in duplicate on different days with freshly prepared substrate and enzyme dilutions. Data were analyzed by non-linear regression of the replicated data. For *p*-coumaroyl- or caffeoyl-CoA donors as the variable substrates with L-Tyr or L-DOPA acceptors as the constant substrates, data were fit with a substrate inhibition kinetics model [Equation 5.44, in Copeland (2000)], whereas all other donor/acceptor substrate combinations were fit with the Michaelis-Menten enzyme kinetics model. Kinetic parameters are reported±standard error (SE) as determined from the non-linear regression.

### Alfalfa Transformation

Plant transformation vectors were introduced into *Agrobacterium tumefaciens* strain LBA4404 by standard methods (Wise et al., 2006). The resulting *A. tumefaciens* strains were used to transform a highly regenerable clone of Regen-SY (Bingham, 1991) as described by Samac and Austin-Phillips (2006).

### Transient Expression of HDT in *N. Benthamiana*

All plasmids used in transient expression experiments were transformed into *A. tumefaciens* strain AGL1 by electroporation (Lazo et al., 1991). *A. tumefaciens* liquid cultures were grown as described by Fister et al. (2016), pelleted by centrifugation (10min at 5,000×*g*), and resuspended in sterile distilled water to OD_600_=0.40±0.01. One volume of cell suspension harboring the p19 silencing suppressor plasmid was mixed with four volumes of those harboring treatment plasmids. Treatments consisted of the empty vector control only (“EV”), HDT1 only (“HDT1”), tyrosine hydroxylase/CYP76AD6 only (“TH”), and HDT1 plus tyrosine hydroxylase (“HDT1+TH”). “TH” and “HDT1” treatments were mixed in equal parts with the empty vector-harboring cell suspension to maintain a consistent cell density of each construct as that used in the “HDT1+TH” co-infiltration.

Leaves were infiltrated with *A. tumefaciens* on the abaxial side using a needleless syringe. Four days after infiltration, successfully transformed leaf disks (indicated by GFP marker gene expression) were collected and extracted as described above.

### Statistical Analyses

For analysis of the whole group of transgenic red clover plants, median and median absolute deviation (MAD) were used as descriptors of the population/subpopulations and statistical significance of differences among groups of plants was assessed by the Wilcoxon-Mann-Whitney rank sum test (Nap et al., 1993). For the experiment comparing transferase activities of the “severe” phenotype and control red clover and for the *N. benthamiana* transient expression experiment comparing empty vector control to treatments, mean and SE were used as descriptors of the data and student’s t-test (Samuels, 1989) was used to assess differences. Real-time PCR data were analyzed by the comparative C_T_ method (Schmittgen and Livak, 2008) using the average C_T_ of three technical replicates for each sample and primer combination. ∆C_T_ was determined by subtracting actin C_T_ from HDT, HST, or HMT C_T_ for each sample. For each plant analyzed, ∆∆C_T_ for each gene was determined by subtracting the mature leaf ∆C_T_ from the unexpanded leave ∆C_T_ and data expressed as mean±SE. Differences between expression levels for mature and unexpanded leaf were evaluated by paired t-test using Prism 8 (GraphPad Software).

## Results and Discussion

### Silencing HMT in Red Clover Reduces Accumulation of Both Phaselic Acid and Clovamide

**Figure 2:**
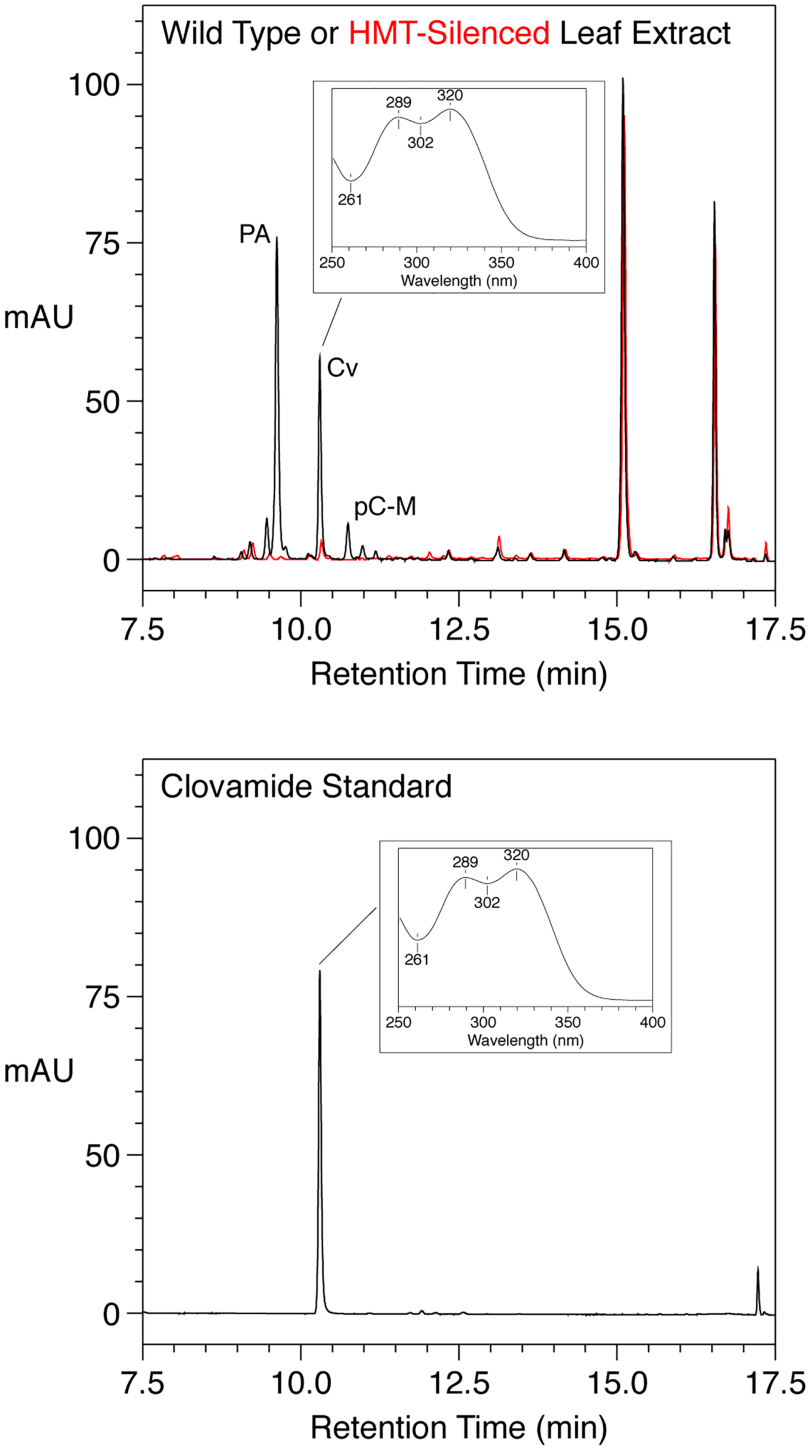
HPLC Separation with PDA detection (250–500nm, absorption in milli absorbance units) of phenolics from mature leaves from a wild type (black) or HMT-silenced (red) red clover plant (top panel) or a purchased clovamide standard (bottom panel). For leaf extracts, *trans*-phaselic acid (PA), -*p*-coumaroyl-malate (pC-M), and -clovamide (Cv) are indicated; major peaks at approximately 15 and 16.5min were not identified but have UV absorption spectra consistent with flavonoids (data not shown). The UV absorption spectrum of the clovamide peak is shown in the insets.

We previously identified and characterized a red clover gene encoding a HMT that is responsible for accumulation of large amounts of phaselic acid in this species (Sullivan, 2009; Sullivan and Zarnowski, 2011). Silencing HMT in red clover by RNAi resulted in reductions of phaselic acid and the related *p*-coumaroyl-malate, but also in an, at the time, unidentified compound (Sullivan and Zarnowski, 2011) in some of the silenced plants. We have subsequently identified this compound as clovamide based on it being indistinguishable from a purchased clovamide standard in terms of its elution behavior from reverse-phase HPLC, UV absorption spectrum, and mass based on MS [(*m/z*) of major ion of −358 and 360 in negative and positive ion mode, respectively; Figure 2].

Among a more recently generated group of red clover plants transformed with an HMT RNAi silencing construct, nine independent transformants carrying the silencing transgene were identified by PCR. Mature leaves of these plants, along with those of three untransformed plants of the same genotype (wild type control), were analyzed for phaselic acid and clovamide accumulation (Table 3). With respect to phaselic acid reduction relative to wild-type plants, the transgenic plants fell into three different groups of three plants each: those whose phaselic acid accumulation was similar to wild type (none), those whose phaselic acid accumulation was approximately 10% of wild type (moderate), and those whose phaselic acid accumulation was approximately 1% or less of wild type (severe). The relatively small sample size makes establishing statistically significant differences here difficult. In fact, using the nonparametric Mann-Whitney test (Nap et al., 1993), no significant difference is seen between the transgenic plants as a whole and wild-type plants with respect to phaselic acid accumulation. However, we previously established a significant correlation between HMT mRNA levels and phaselic acid accumulation (Sullivan and Zarnowski, 2011). Further, it seems likely that among the plants with moderate and severe reductions in phaselic acid accumulation, these reductions are almost certainly due to the presence of the HMT silencing transgene since among a number of analyses of phaselic acid in red cover leaves, levels this low have never been reported except when HMT has been downregulated (Winters et al., 2008; Saviranta et al., 2010; Sullivan and Zeller, 2013). Similar to our previous observation, some of the transgenic plants also had reductions in clovamide accumulation. Interestingly, only plants with severe reductions in phaselic acid accumulation had substantial reductions (to about 10% wild-type levels) in clovamide. This reduction, compared to wild type, was significant by the Mann-Whitney test at *p*=0.10, the lowest *p*-value possible with this small sample size. Given that the HMT enzyme does not itself have hydroxycinnamoyl-CoA:L-DOPA hydroxycinnamoyl transferase activity (Sullivan, 2009), the reduction in clovamide levels resulting from HMT silencing suggested that a gene encoding a transferase responsible for clovamide biosynthesis shares sufficient nucleotide sequence identity with HMT to be downregulated by the HMT RNAi construct.

**Table 3:** *trans*-Phaselic acid and *trans*-clovamide content^a^ in mature leaves for groups of plants transformed with an HMT RNAi construct.

Phaselic acid reduction^b^	n^c^	Phaselic acid content	Clovamide content
NA (wild type)	3	10.0 ± 1.1	2.8 ± 0.1
None	3	12.5 ± 1.2	3.4 ± 0.6
Moderate	3	0.8 ± 0.1	2.2 ± 0.5
Severe	3	0.1 ± 0.0	0.3 ± 0.1

a Median±median absolute deviation in μmol/g fresh weight.

b Relative to wild type; NA: not applicable.

c n corresponds to the number of independent transgenic events analyzed, except for wild type, which were individual clones of NRC7 genotype.

### Evaluation of Red Clover Leaves for and Preliminary Characterization of HDT Activity

Leaf extracts were prepared as previously described (Sullivan, 2009) and incubated with caffeoyl-CoA and L-DOPA to assess HDT activity in red clover leaves. In these experiments with crude extracts, we found HDT activity, leading to the formation of clovamide from caffeoyl-CoA and L-DOPA, was highest in 100mM sodium phosphate buffer at pH 7.5, the highest pH tested. HDT activity was readily detected in unexpanded young leaves (on the order of 10pkat/mg crude protein) but undetectable in fully expanded mature leaves. Subsequent experiments with purified protein, including kinetics experiments, showed higher HDT activity at pH 8.0 (Sullivan and Bonawitz, 2018 and as detailed below). pH conditions above 8.0 were not tested due to sensitivity of CoA thioester linkages to alkaline conditions.

We further assessed HMT, HDT, and HST activity from unexpanded leaves of the three plants transformed with the HMT RNAi construct that showed the most dramatic reductions in phaselic acid (and clovamide) content (“Severe” in Table 3) as well as four plants (each an independent event) transformed with the corresponding empty binary transformation vector (Table 4). As expected, in the plants transformed with the HMT RNAi construct that failed to accumulate phaselic acid, HMT activity was reduced to undetectable levels compared with the control (empty vector plants). For HDT, activity was significantly reduced about 20-fold relative to the vector control plants. Although hydroxycinnamoyl-shikimate esters do not accumulate to substantial levels in red clover leaves, relatively high levels of HST activity are present in red clover leaves (Sullivan, 2009), presumably to provide the *p*-coumaroyl-shikimate intermediate for biosynthesis of caffeic acid moieties (Schoch et al., 2006). For HST, activity was not reduced, and in fact significantly increased approximately 1.5-fold. Because the HST coding region shares only 54% identity with that of HMT (and no stretches of the approximately 20 nucleotides of identity typically needed for RNAi - mediated silencing), it is not surprising that transferase activity was not reduced in the HMT-silenced plants. It is unclear why there was a modest increase in transferase activity, but this increase could reflect upregulation in response to the decreased accumulation of phaselic acid and clovamide or changes in hydroxycinnamoyl-CoA pools due to the HMT silencing.

**Table 4:** Hydroxycinnamoyl-CoA transferase activities^a^ in extracts of unexpanded leaves of control and HMT-silenced (“Severe” phenotype, Table 3) red clover plants.

Construct	n^b^	HMT	HDT	HST
Empty Vector	4	4.9 ± 0.32^*^	9.5 ± 2.10^*^	65.0 ± 5.87^*^
HMT Silencing	3	0.0 ± 0.00^*^	0.5 ± 0.11^*^	90.1 ± 7.85^*^

a Activities expressed as pkat/mg crude protein±standard error using the following donor/acceptor pairs: HMT, p-coumaroyl-CoA/malic acid; HDT, caffeoyl-CoA/L-DOPA; and HST, p-coumaroyl-CoA/shikimic acid.

b n corresponds to independent transgenic events.

* Values within columns are significantly different at *p*<0.05.

### Cloning of a cDNA Encoding HDT

Given that the data described above suggested HDT was encoded by a BAHD hydroxycinnamoyl transferase with sequence similarity to red clover HMT, degenerate oligonucleotide PCR primers were designed based on several conserved regions of red clover HMT and HST, and two additional likely BAHD hydroxycinnamoyl transferases from *Phaseolus vulgaris* (see Materials and Methods). These primers were used in nested PCR reactions with cDNA prepared from unexpanded red clover leaves to generate an approximately 700bp DNA fragment. Sequence analysis of the fragment revealed that it was distinct (71–81% identity) from the red clover HMT cDNA sequence as well as those of two other previously identified red clover genes likely encoding BAHD hydroxycinnamoyl-CoA hydroxycinnamoyl transferases (GenBank MW605157, MW605158). Like HMT, these had been ruled out as being involved in clovamide biosynthesis based on lack of detectable enzyme activity for protein expressed in *E. coli*. Based on the fragment sequence, we were able to design primers for 5′ and 3′ RACE. The resulting RACE products were sequenced and used to design primers for end-to-end PCR to generate full-length clones corresponding to the putative HDT cDNA. For end-to-end PCR, a high fidelity proofreading thermostable DNA polymerase was used and clones were isolated and sequenced from three independent PCR reactions allowing authentic alleles of the putative HDT gene to be distinguished from PCR errors (true alleles would be expected to be represented in all three independent PCR reactions, whereas this would be unlikely for PCR-induced base changes).

**Figure 3:**
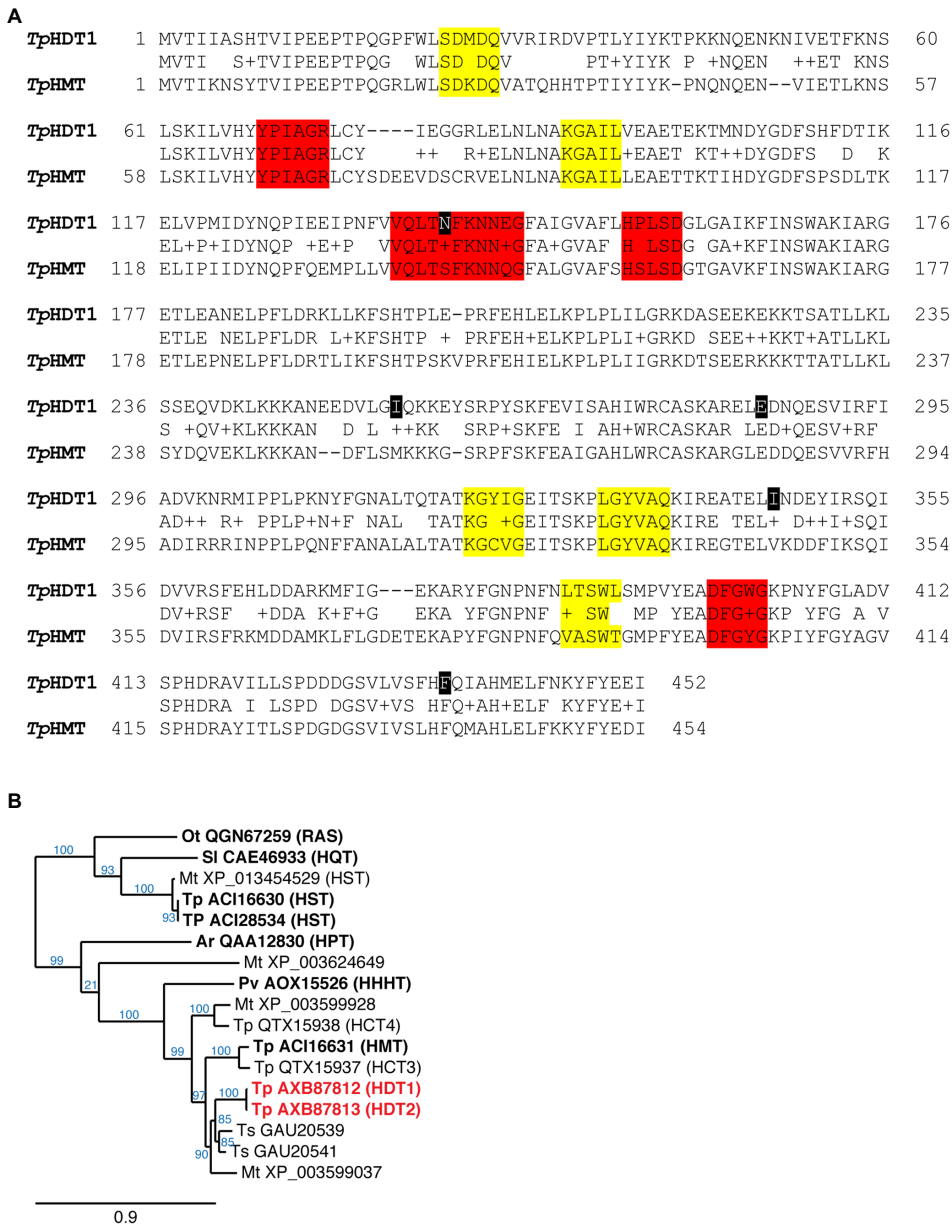
**(A)** Amino acid sequence comparison of red clover HDT1 and HMT. Gaps are shown with a “-” and conservative substitutions are shown with a “+.” Amino acids that differ between HDT1 and HDT2 are marked in black (K140N, V255I, K285E, V346I, and L436F). Motifs conserved among all BAHD acyltransferases are highlighted in red while motifs specific to the hydroxycinnamoyl transferase-containing Clade Vb are highlighted in yellow (D'Auria, 2006; Tuominen et al., 2011). **(B)** Phylogenetic relationship of red clover HDT to several other Clade Vb BAHD acyltransferases, which contains hydroxycinnamoyl-CoA hydroxycinnamoyl transferases. Dendrogram was generated with BAHD hydroxycinnamoyl-CoA hydroxycinnamoyl transferase amino acid sequences as described in Materials and Methods. For each protein sequence, the GenBank identifier is provided and species from which the sequences are derived are designated as follows: Ar, *Actaea racemosa*; Mt., *Medicago truncatula*; Ot, *Ocimum tenuiflorum*; Pv, *Phaseolus vulgaris*; Sl, *Solanum lycopersicum*; Tp, *Trifolium pratense*; and Ts, *Trifolium subterraneum*. HDT1 and 2 are highlighted in red. Transferases that have been biochemically characterized are in bold text. Enzyme activities (in parentheses) are hydroxycinnamoyl-CoA hydroxycinnamoyl transferases with the following acceptor substrates: HDT, L-DOPA/L-Tyr; HMT, malate; HHHT, tetrahydroxyhexanedioic acid; HPT, piscidic acid; RAS (rosmarinic acid synthase), hydroxyphenyllactate; HST, shikimic acid; and HQT, quinic acid. Branch support values (%) are shown in blue.

Using this approach, two distinct 1,451bp cDNAs were identified. These were designated HDT1 and HDT2 (deposited in GenBank as MF115997 and MF115998, respectively; nucleotide and corresponding amino acid sequence of HDT1 is shown in Supplemental Figure S1). Nucleotide sequences of the cDNAs are >99% identical and are predicted to encode 452 amino acid proteins that differ by 5 amino acid residues (>98% identical; Figure 3A). Nucleotide sequences of the HDT open reading frames are 80% identical to that of red clover HMT and include multiple stretches ≥21 nucleotides of perfect identity or containing only a single mismatched base as might be required for off-target silencing by the HMT RNAi construct.

Compared to red clover BAHD hydroxycinnamoyl-CoA transferases, the predicted encoded HDT proteins are 72% identical to HMT (Figures 3A,B), 71% identical to QTX15937 (encoded by GenBank MW605157), and 67% identical QTX15938 (encoded by GenBank MW605158), but only 35% identical to that of red clover HST. The most similar proteins from species other than red clover are from legumes and include GAU20541 (82% identical) and GAU20539 (80% identical) from *Trifolium subterraneum* and XP_003599037 (75% identical) from *Medicago truncatula*, none of which have been biochemically characterized. Amino acid motifs characteristic of BAHD acyltransferases in general (D'Auria, 2006) and BAHD hydroxycinnamoyl transferases specifically (Tuominen et al., 2011) are apparent in the red clover HDT proteins (Figure 3A) and phylogenetic analysis places them in Clade Vb [as defined by Tuominen et al. (2011)] with other enzymatically characterized or proposed BAHD hydroxycinnamoyl-CoA transferases (Figure 3B).

### HDT mRNA Accumulation Corresponds to HDT Activity in Red Clover

We used quantitative real-time PCR to evaluate HDT mRNA accumulation in red clover leaves. cDNA was prepared from young unexpanded or mature leaves from five different wild-type red clover plants and subjected to real-time PCR using primers specific to HDT. Expression of HST and HMT was also evaluated. In all cases, expression was normalized to that of actin. As shown in Table 5, HDT expression was approximately 60-fold higher in unexpanded leaves relative to mature leaves. The low mRNA expression for HDT in mature leaves relative to unexpanded leaves is consistent with our failure to detect HDT activity in mature leaves. Expression levels for HST were indistinguishable between unexpanded and mature leaves, and HMT expression levels seemed slightly higher (approximately 2-fold) in mature leaves relative to unexpanded leaves, although the difference was not significant at *p*<0.05. These findings are consistent with our previous analysis of HST and HDT expression in these tissues (Sullivan, 2009). Unfortunately, red clover plants silenced for HMT (or RNA from their unexpanded leaves) were no longer available for analysis to confirm that HDT mRNA levels were reduced in those plants relative to wild type.

**Table 5:** Real-time PCR analysis of expression of HDT, HST, and HMT in unexpanded and mature red clover leaves.

Gene	Log_2_-Fold Change	*P*-value^a^
	[∆∆C_T_ (unexpanded-mature)]	
HDT	−5.93 ± 1.50	0.02
HST	0.05 ± 0.16	0.77
HMT	1.14 ± 0.45	0.07

a From paired t-test, mature versus unexpanded.

### Expression of HDT in *E. Coli* and Characterization of the Encoded Protein

**Figure 4:**
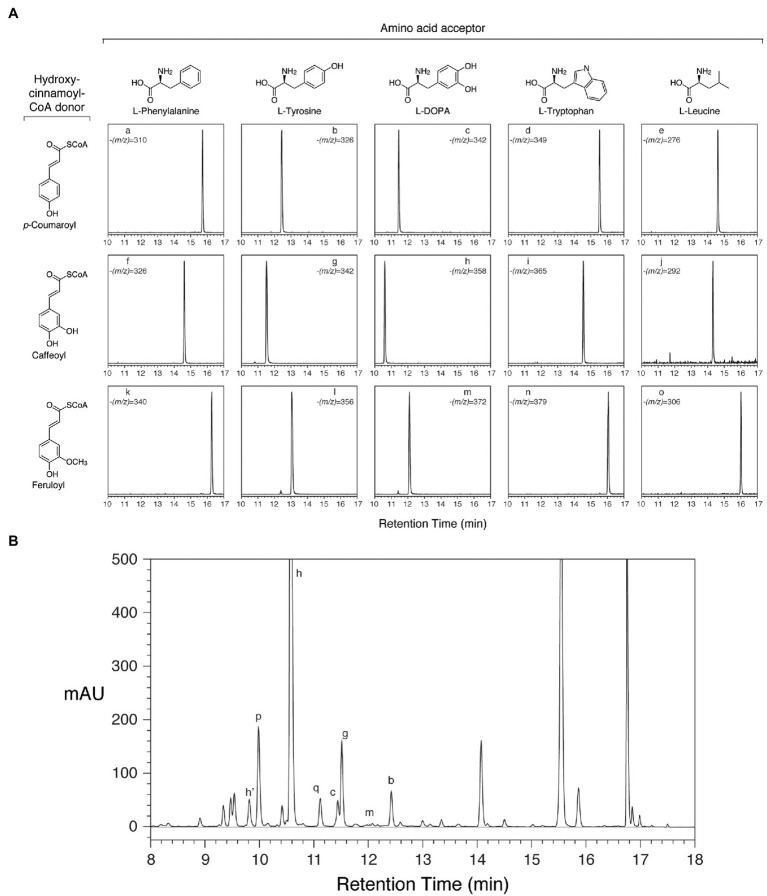
Reaction products from HDT expressed in *Escherichia coli* correspond to phenolic compounds in red clover leaves. Peak assignments are detailed in Table 6. **(A)** HPLC separation and selective ion MS detection of *in vitro* HDT reaction products of the indicated donor and acceptor substrates. The (*m/z*) value monitored in each chromatogram is indicated. **(B)** HPLC separation of phenolics extracted from unexpanded red clover leaves with PDA (250–500nm) detection.

To determine whether the cloned red clover cDNAs encode enzymes with hydroxycinnamoyl-CoA:L-DOPA hydroxycinnamoyl transferase activity, the open reading frames were placed behind the IPTG-inducible promoter of the pET28 expression vector and expressed in *E. coli* (Sullivan and Bonawitz, 2018). Crude *E. coli* extracts containing the recombinant proteins were incubated with hydroxycinnamoyl-CoAs and acceptors, and the reactions were analyzed by HPLC with PDA and MS detection. The protein products of the cloned HDT cDNAs were capable of transferring a hydroxycinnamoyl moiety from *p*-coumaroyl-, caffeoyl-, and feruloyl-CoA to L-DOPA, L-Tyr, or L-Phe based on accumulation of products with the expected *m/z* as determined by MS (shown for HDT1 in Figure 4). In the case of caffeoyl-L-Tyr and caffeoyl-L-DOPA, where authentic compound was available for purchase, the *in vitro*-formed products were indistinguishable from the purchased standards in terms of retention time, UV absorption spectrum, and (*m/z*). For caffeoyl-L-Tyr, we also produced sufficient compound *via* an HDT1-mediated reaction to carry out ^1^H and ^13^C NMR to further confirm product identity (Supplementary Figure S2). Chemical shifts were consistent with those previously published for this compound (Stark and Hofmann, 2005). In all cases, product formation required the presence of the recombinant protein, as control extracts of *E. coli* transformed with pET28 lacking an HDT insert failed to form these amide products when incubated with hydroxycinnamoyl-CoA donor and acceptor substrates. In addition to the above aromatic amino acids, L-Trp and L-Leu were also tested as acceptors as it had been demonstrated that HDT1 substrate specificity might extend to these amino acids as well based on HDT1 expression in a novel yeast system (Bouchez et al., 2019). Although not all amino acids were tested as acceptors, no product was detected when arginine was used as an acceptor. Further, Bouchez et al. (2019) were unable to find evidence of other amino acid acceptors for HDT1 (beyond L-DOPA, L-Tyr, L-Phe, L-Trp, and L-Leu), suggesting specificity for aromatic, and perhaps some hydrophobic (e.g., L-Leu), amino acids. We only tested L-amino acids with HDT1, since this is what is present in clovamide and what is most abundantly present in nature. Interestingly, Sander and Petersen (2011) showed rosmarinic acid synthase from *Coleus blumei* is capable of forming hydroxycinnamoyl amides with D-Phe, D-Tyr, and D-DOPA. In this case, ability of the D-amino acid substrates to serve as acceptor substrates could be due to their structural (including stereochemistry) similarity to the D-phenyllactic acid acceptor also used by this enzyme. For HDT1, no reaction products were seen when tyramine was used as an acceptor, suggesting that the carboxyl moiety of the amino acid acceptors is important for recognition as a substrate. No reaction products were seen when malic acid, the acceptor for red clover HMT, was used as an acceptor in the reactions.

Besides clovamide (peak h), the separation behavior of the *in vitro*-produced compounds by reverse-phase HPLC allowed the identification of three additional *N*-hydroxycinnamoyl amides in unexpanded red clover leaves including *p*-coumaroyl amides with both L-Tyr (peak b) and L-DOPA (peak c), and caffeoyl-L-Tyr (peak g; Figure 4B and Table 6). A very minor peak at approximately 12.0min (peak m) was consistent with feruloyl-L-DOPA based on retention time, λ_max_, and MS signal. Other amides with ferulic acid were not readily detected in unexpanded red clover leaves by either PDA or MS. Although there was no obvious peak detected by PDA, we detected what might be very small amounts of caffeoyl-L-Phe (based on coelution of MS signal with product prepared *in vitro*) in unexpanded red clover leaves (none detected in mature leaves), with a signal several 100-fold lower than that detected for clovamide. No other hydroxycinnamoyl-L-Phe amides (based on expected elution time and MS signal) were detected in either unexpanded or mature red clover leaves nor were L-Trp or L-Leu amides detected *in vivo*, despite ability of HDT to synthesize these *in vitro*. Additionally, HPLC traces from Figures 2, 4 are typical and reveal differences in relative abundances of phaselic acid and clovamide, at least in this genotype. We have consistently observed that clovamide is more abundant than phaselic acid in unexpanded leaves [22.6±0.7 versus 4.4±0.2μmol/g FW (median±MAD), respectively], whereas phaselic acid is more abundant than clovamide in mature leaves (Table 3). This observation is consistent with the substantial decline of HDT expression in mature leaves compared to unexpanded leaves, while HMT expression has a modest increase in mature leaves compared to unexpanded leaves. That substantial amounts of clovamide persist in mature leaves despite a lack of HDT activity suggests the compound is fairly stable *in vivo*. It is unclear whether this shift in caffeic acid derivatives from young leaves to matures leaves has any biological role. However, clovamide accumulation has been observed to vary among different genotypes (Sullivan and Zeller, 2013) and throughout the growing season under field conditions (Winters et al., 2008), and to increase in response to jasmonic acid treatment (Tebayashi et al., 2000) suggesting perhaps some unique biological functions for it.

**Table 6:** Peak characteristics and identities from Figure 4.

Peak	T_R_ (min)	λ_max_ (nm)	(M-H)^a^	Identity
a	15.7	308	310	*trans*-*p*-coumaroyl-L-Phe
b	12.4	308	326	*trans*-*p*-coumaroyl-L-Tyr
c	11.4	291/308	342	*trans*-*p*-coumaroyl-L-DOPA
d	15.5	290/307	349	*trans*-*p*-coumaroyl-L-Trp
e	15.4	309	276	*trans*-*p*-coumaroyl-L-Leu
f	14.6	326	326	*trans*-caffeoyl-L-Phe
g	11.5	296/320	342	*trans*- caffeoyl-L-Tyr
h	10.6	290/320	358	*trans*- caffeoyl-L-DOPA (clovamide)
i	14.5	290/323	365	*trans*-caffeoyl-L-Trp
j	14.3^b^	ND^b^	292	*trans*-caffeoyl-L-Leu
k	16.2	320	340	*trans*-feruloyl-L-Phe
l	13.0	319	356	*trans*-feruloyl-L-Tyr
m	12.0	290/319	372	*trans*-feruloyl-L-DOPA
n	16.0	290/320	379	*trans*-feruloyl-L-Trp
o	16.0	319	306	*trans*-feruloyl-L-Leu
p	10.0	327	295	*trans*-caffeoyl-malate (phaselic acid)
q	11.1	313	279	*trans-p*-coumaroyl*-malate*
h’	9.8	291	358	*cis*-caffeoyl-L-DOPA (tentative)

a Molecular weight of major negative ion detected.

b PDA peak was not well separated from another peak, so T_R_ was estimated from the MS peak T_R_ and *λ*_max_ could not be determined.

### Determination of HDT Kinetic Parameters

For a more detailed analysis of the enzymatic properties of HDT, a histidine-tagged, codon-optimized version of HDT1 was expressed in *E. coli* and purified by metal affinity chromatography (Sullivan and Bonawitz, 2018) and the resulting protein was used in kinetic analyses of *trans*-*p*-coumaroyl-, -caffeoyl-, and -feruloyl-CoA donor and L-Tyr, L-DOPA, and L-Trp acceptor substrates. More limited analyses (explained below) were carried out with L-Phe and L-Leu acceptor substrates. The results of this analysis are shown in Table 7 and Supplementary Figure S3.

**Table 7:** Kinetic parameters for red clover HDT1.

Variable Substrate^a^	Constant Substrate^a^	*K*_M_ (μM)	*K*_i_ (μM)	*k*_cat_ (s^−1^)	*k*_cat_/*K*_M_ (s^−1^ μM^−1^)
*p*-CA-CoA	L-Tyr (3,000μM)	2.96 ± 0.320	72.5 ± 11.40	3.45 ± 0.164	1.17 ± 0.138
CA-CoA	L-Tyr (3,000μM)	7.37 ± 0.831	292.2 ± 63.64	10.17 ± 0.481	1.38 ± 0.169
FA-CoA	L-Tyr (3,000μM)	0.72 ± 0.081	–	0.76 ± 0.013	1.07 ± 0.122
*p*-CA-CoA	L-DOPA (10,000μM)	2.47 ± 0.576	50.5 ± 14.08	2.72 ± 0.267	1.10 ± 0.278
CA-CoA	L-DOPA (10,000μM)	5.08 ± 0.696	368.4 ± 97.90	5.99 ± 0.325	1.18 ± 0.174
FA-CoA	L-DOPA (10,000μM)	0.64 ± 0.093	–	0.75 ± 0.015	1.16 ± 0.169
*p*-CA-CoA	L-Trp (10,000μM)	0.30 ± 0.031	–	0.82 ± 0.011	2.76 ± 0.286
CA-CoA	L-Trp (10,000μM)	1.29 ± 0.158	–	1.07 ± 0.027	0.83 ± 0.104
FA-CoA	L-Trp (10,000μM)	0.34 ± 0.029	–	0.37 ± 0.004	1.08 ± 0.091
L-Tyr	*p*-CA-CoA(20μM)	145.3 ± 5.56	–	2.74 ± 0.027	0.0189 ± 0.00075
L-Tyr	CA-CoA (50μM)	342.0 ± 14.37	–	9.12 ± 0.114	0.0267 ± 0.00117
L-Tyr	FA-CoA (20μM)	14.4 ± 0.64	–	0.69 ± 0.006	0.0479 ± 0.00216
L-DOPA	*p*-CA-CoA(20μM)	989.8 ± 35.92	–	2.00 ± 0.022	0.0020 ± 0.00008
L-DOPA	CA-CoA (50μM)	2465.0 ± 65.88	–	6.46 ± 0.325	0.0026 ± 0.00008
L-DOPA	FA-CoA (20μM)	150.0 ± 7.65	–	0.75 ± 0.015	0.0050 ± 0.00026
L-Trp	*p*-CA-CoA(20μM)	2163.0 ± 63.39	–	1.00 ± 0.009	0.0005 ± 0.00001
L-Trp	CA-CoA (50μM)	1252.0 ± 26.69	–	1.21 ± 0.007	0.0010 ± 0.00002
L-Trp	FA-CoA (20μM)	273.2 ± 24.15	–	0.35 ± 0.007	0.0013 ± 0.00012
L-Phe^b^	CA-CoA (50μM)	>10,000	ND	ND	ND
L-Leu^b^	CA-CoA (50μM)	>10,000	ND	ND	ND

a Variable and constant substrates are as: *p*-CA-CoA, *p*-coumaroyl-CoA; CA-CoA, caffeoyl-CoA; FA-CoA, feruloyl-CoA; L-Tyr, L-tyrosine; L-DOPA, L-3,4-dihydroxyphenlylalanine; L-Trp, L-tryptophan; L-Phe, L-phenylalanine; and L-Leu, L-leucine. The concentration of the constant substrate used is shown in parentheses.

b ND, not determined.

Considering first reactions involving the structurally similar acceptors L-Tyr and L-DOPA, *K*_M_ values for a given donor substrate were similar and had the same rank order among the other donors regardless of whether L-Tyr or L-DOPA was the acceptor substrate. For the donor substrates, turnover number (*k*_cat_) varied directly with *K*_M_ so that catalytic efficiencies (as *k*_cat_/*K*_M_) were roughly similar for all the donor and acceptor substrate combinations tested. Both *p*-coumaroyl- and caffeoyl-CoA donor substrates exhibited substrate inhibition, with *K*_i_ values of around 70 and 300μM, respectively. Substrate inhibition was not apparent with feruloyl-CoA under the conditions tested, suggesting either that this substrate does not inhibit the reaction or the *K*_i_ for this substrate is so high as to have little impact on the reaction at the concentrations tested.

Because of the substrate inhibition seen with the *p*-coumaroyl- and caffeoyl-CoA donors, for determination of acceptor substrate kinetic parameters, *p*-coumaroyl- and caffeoyl-CoA were used at 20 and 50μM, respectively, the concentrations which gave maximal reaction velocities. Feruloyl-CoA was used at 20μM, a concentration greater than 20-fold higher than the measured *K*_M_ for either the L-Tyr or L-DOPA acceptor. For a given donor, L-Tyr had *K*_M_ values that were 5- to 10-fold lower than those of L-DOPA. *K*_M_ values for the acceptor substrates did depend on donor substrate, being lowest when feruloyl-CoA was the donor and highest when caffeoyl-CoA was the donor. No substrate inhibition was observed for the acceptor substrates at the concentrations tested. Turnover numbers were similar to those measured with varying donor substrate. The net effect was that catalytic efficiency is about 10-fold higher for L-Tyr as an acceptor compared to L-DOPA.

Considering reactions involving L-Trp as an acceptor substrate, no donor substrate inhibition was apparent and *K*_M_ values for a given donor substrate were substantially lower and had a different rank order than those measured for L-Tyr or L-DOPA. *k*_cat_ values were also substantially lower than those seen when L-Tyr or L-DOPA was used as acceptors, with the net effect of overall catalytic efficiencies being in a similar range as seen for L-Tyr and L-DOPA acceptors, although varying over a wider range. For determination of acceptor substrate kinetic parameters, *p*-coumaroyl-, caffeoyl-, and feruloyl-CoA were used at 20, 50, and 20μM, respectively, the same concentrations used for the measurements with L-Tyr and L-DOPA and >30-fold over any of the measured *K*_M_ for any of these donors. For a given donor, L-Trp had *K*_M_ values that were 3- to 20-fold higher than those of L-Tyr, but in a similar range as those seen for L-DOPA (within about 2-fold). *K*_M_ values for this acceptor substrate did depend on donor substrate, being lowest when feruloyl-CoA was the donor (as for L-Tyr and L-DOPA) and highest when *p*-coumaroyl-CoA was the donor (unlike what was seen for L-Tyr and L-DOPA). Turnover numbers were similar to those measured with varying donor substrate. The net effect was that catalytic efficiencies are 20- to 40-fold and 2- to 4-fold lower than those seen for L-Tyr and L-DOPA, respectively.

Some preliminary measurements were carried out to assess kinetic parameters with L-Phe and L-Leu as acceptors. It was apparent from these preliminary rate measurements that the *K*_M_ values for L-Phe and L-Leu, at least with caffeoyl-CoA as donor, are >10,000μM. Given the practical difficulties of using such high substrate concentrations for kinetic measurements, the uncertain physiological relevance of such high *K*_M_ values, and the lack of substantial accumulation of hydroxycinnamoyl-L-Phe or -L-Leu products *in vivo*, no further kinetic measurements were made using these as acceptors.

Kinetic analyses showed that for donor substrates, catalytic efficiency is similar for the tested hydroxycinnamoyl-CoAs since *K*_M_ and turnover numbers, for the most part, varied directly. Thus, *in vivo*, accumulation of specific hydroxycinnamoyl products may reflect donor substrate availability. In the case of red clover HMT, caffeoyl-CoA appears to not be the favored donor substrate, yet the caffeoyl-malate product (phaselic acid) predominates (Sullivan and Zarnowski, 2011; Sullivan and Zeller, 2013). Although caffeoyl products could be the result of 3′ hydroxylation of *p*-coumaroyl-malate, we have been unable to identify a hydroxylase with this activity in red clover (Sullivan and Zarnowski, 2010). The possible reasons for caffeoyl products predominating in red clover may be similar for HDT.

For acceptor substrates, the *K*_M_ value for a given acceptor varied widely (up to >20-fold) depending on the donor substrate with which it was being tested, although catalytic efficiency for a given acceptor varied by less than 3-fold because, as for donor substrates, *K*_M_ and *k*_cat_ for the most part varied directly. Interestingly, catalytic efficiency was around 10-fold higher for L-Tyr than for L-DOPA, suggesting the possibility that *in vivo*, clovamide is made *via* hydroxylation of the L-Tyr moiety of caffeoyl-L-Tyr. Recent work in plants indicates at least two pathways whereby L-DOPA moieties are made, one involving PPO (Araji et al., 2014) and another involving cytochrome P450 enzymes (Sunnadeniya et al., 2016). It is not known how L-DOPA moieties are made in red clover, but silencing of the major PPO genes in red clover does not appear to reduce levels of clovamide ((Webb et al., 2014) and additional unpublished data), suggesting another pathway, perhaps involving a cytochrome P450, acting on either free L-Tyr or the L-Tyr moiety of a hydroxycinnamoyl-L-Tyr amide. Given that HDT appears to favor use of L-Tyr over L-DOPA, if L-DOPA moieties are formed from L-Tyr and used for clovamide biosynthesis, substrate channeling might be required to favor L-DOPA utilization. The analysis of L-Trp as acceptor showed poor catalytic efficiency compared to L-Tyr (>25-fold lower for all CoA donors tested). A limited analysis using L-Phe and L-Leu as acceptors suggests they also are poor acceptors for HDT with an extremely high *K*_M_, and, especially given the lack of *in vivo* products containing L-Trp, L-Phe, or L-Leu moieties in either red clover or transgenic alfalfa expressing HDT (described below), these likely are not physiologically relevant substrates.

*p*-Coumaroyl- and caffeoyl donors both exhibited substrate inhibition when used with L-Tyr or L-DOPA as acceptors. Feruloyl-CoA did not appear to inhibit the reaction, or, if it does, only weakly such that inhibition was not apparent at the substrate concentrations tested. Inhibition by the CoA donor substrate has been reported for other BAHD family acyltransferases (see, for example, Schmidt et al., 2014). In the case of HDT, donor substrate inhibition could be due to the structural similarity of these donors to the L-Tyr and L-DOPA acceptor substrates (i.e., both donor and acceptor contain a phenolic ring). Thus, it may be that the *p*-coumaroyl- and caffeoyl-CoA substrates are able to effectively compete for the acceptor substrate binding site on the enzyme, while perhaps the bulky methoxy group on feruloyl-CoA prevents this competition. This could also explain why acceptor *K*_M_ is substantially lower with feruloyl-CoA as the donor: Less acceptor would be required to saturate its binding site in the absence of an effective competitor. Interestingly, neither L-Tyr nor L-DOPA induce similar substrate inhibition, or, if they do, the *K*_i_ of such inhibition is much higher than the acceptor substrate concentrations tested here.

To examine some of these ideas about the relationship of enzyme structure to function (e.g., whether hydroxycinnamoyl-CoA donors might be competing with acceptors at the acceptor binding site), we did attempt protein structure homology modeling using SWISS-MODEL (Biasini et al., 2014) with both the *Sorghum bicolor* HST (Walker et al., 2013) and the *Coffea* hydroxycinnamoyl-CoA:quinate hydroxycinnamoyl transferase (Lallemand et al., 2012) as templates. Unfortunately, the resulting models were predicted to be of very low quality with QMEN scores below −4, presumably because of the relatively low amino acid sequence identity (35%) shared by HDT and these hydroxycinnamoyl transferases for which structures have been determined.

### Stable Transgenic Expression of HDT in Alfalfa

**Figure 5:**
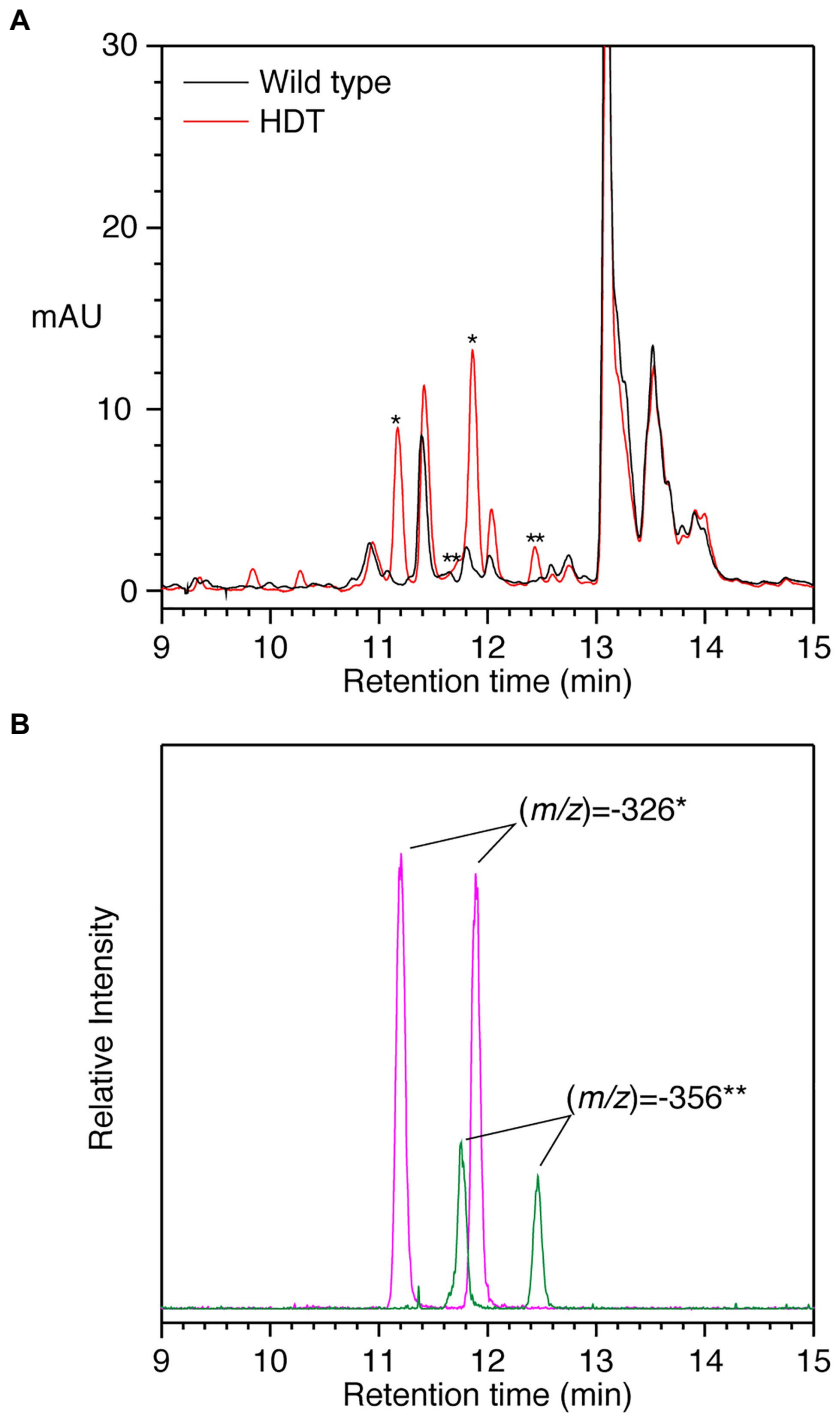
HPLC separation of phenolics from alfalfa transformed with HDT. **(A)** PDA detection (250–500nm) of phenolics from wild type (black) or HDT-expressing (red) alfalfa. Asterisks denote peaks present in the transgenic alfalfa with MS signals expected for *p*-coumaroyl- and feruloyl-L-Tyr, ^*^ and ^**^, respectively. **(B)** Ion selective chromatograms of the transgenic plant phenolic separation shown in Panel A. Signal corresponding to (*m/z*) of −326 (magenta) and −356 (green) is shown. These signals were not present in alfalfa transformed with vector only. Amides composed of caffeoyl and L-DOPA moieties were not detected based on MS signals. Retention times are slightly shorter compared to Figures 2, 4 due to differences in HPLC configuration between the experiments.

To further assess the function of HDT, both HDT cDNAs were placed under the control of the strong constitutive CsVMV protomer (Samac et al., 2004) and transformed into alfalfa, which does not accumulate significant amounts of hydroxycinnamoyl esters or amides in its leaves (Sullivan, 2017). Eight independent transformants harboring the HDT1 transgene and one transformant harboring the HDT2 transgene were recovered based on PCR screening of genomic DNA. Leaf extracts of all the transgenic plants were analyzed by HPLC with PDA and MS detection. Extracts from five of the eight independent HDT1 transformants and the HDT2 transformant had new peaks compared to wild-type control plants. A typical chromatogram is shown in Figure 5 for one of the HDT1 plants. The HDT2 plant was indistinguishable from the HDT1 plants in terms of new peaks present. Analysis by MS showed the most prominent new peaks associated with expression of HDT had (*m/z*) of −326 and−356. The peak of (*m/z*) of −326 eluting at 11.9min is almost certainly *trans*-*p*-coumaroyl-L-Tyr as it has the same elution behavior, (*m/z*), and absorption spectrum as a compound synthesized *in vitro* by HDT expressed in *E. coli* incubated with *trans*-*p*-coumaroyl-CoA and L-Tyr. The peak of (*m/z*) of −326 eluting at approximately 11.2min is presumably *cis*-*p*-coumaroyl-L-Tyr. Unfortunately, unlike some other hydroxycinnamoyl compounds (Sullivan, 2014, 2017), we have been unable to reliably interconvert the *trans*- and *cis*-versions of clovamide and related hydroxycinnamoyl-aromatic amino acid amides by UV treatment *in vitro*. However, that this earlier eluting compound is the *cis*-version is supported by the observation that in planta, both *trans* and *cis*-versions of accumulating hydroxycinnamoyl compounds tend to be present (Sullivan, 2014, 2017). Further, over time, stock solutions of purchased pure *trans*-clovamide or *trans*-caffeoyl-L-Tyr accumulate an earlier eluting compound of the same molecular weight based on MS. In the case of clovamide, the earlier eluting compound derived from the purchased pure compound has identical elution, UV absorption spectrum, and molecular weight as a compound found in red clover leaves (peaks h and h’ in Figure 4 and Table 6). These findings suggest that for clovamide and related compounds (i.e., *N*-hydroxycinnamoyl amides with phenolic amino acids), the *cis*-version elutes earlier than the *trans* version under our chromatography conditions. Similarly, the peak with (*m/z*) of −356 eluting at approximately 12.5 and the small peak at 11.8min are most likely *trans*- and *cis*-feruloyl-L-Tyr, respectively, as the 12.5min peak exhibits elution and spectral behaviors indistinguishable from the compound formed by incubating purified HDT with *trans*-feruloyl-CoA and L-Tyr.

Notably, no MS signals were detected for (*m/z*) of −342 (expected for *p*-coumaroyl-L-DOPA or caffeoyl-L-Tyr) or −358 (clovamide). MS signals for (*m/z*) of −372 (expected value for feruloyl-L-DOPA) were detected, but these were minor and coeluted with the (*m/z*) of −326 peaks and likely represent adducts with formic acid present in the HPLC solvent. Although a peak at approximately 12.05min was enhanced in the transgenic alfalfa, it does not appear to differ from that present in wild-type plants with respect to UV absorption spectrum or MS signals. Peaks present in some of the transgenic plants at approximately 9.8 and 10.3min could not be identified based on UV absorption spectra nor MS signals. Thus, it seems that in alfalfa, *p*-coumaroyl and feruloyl amides with L-Tyr are the predominant accumulating products in plants expressing the red clover HDT gene. We have observed a similar lack of accumulation of caffeoyl derivatives when red clover HMT (Sullivan et al., 2021) or bean HHHT (Sullivan, 2017) are expressed in alfalfa. Although the *K*_M_ value of HDT1 for feruloyl-CoA is lower than that of *p*-coumaroyl- or caffoyl-CoA for transfer to L-Tyr or L-DOPA, overall catalytic efficiencies are similar. Thus, it may be the case that the pool of caffeoyl-CoA is limiting in alfalfa leaves. Similarly, L-DOPA acceptor may be lacking in alfalfa while present in red clover. Alternatively, it could be that in red clover, the L-DOPA moiety in hydroxycinnamoyl-L-DOPA amides is formed by 3-hydroxylation of the corresponding hydroxycinnamoyl-L-Tyr amide by an as yet unidentified enzyme not present in alfalfa. Given that L-Tyr appears to be a better acceptor substrate than L-DOPA (with both a lower *K*_M_ and a higher catalytic efficiency), hydroxylation of L-Tyr following amide formation is an attractive model. Additional work is needed to determine how L-DOPA moieties are synthesized in red clover.

Although we had no purified standards available for quantifying the *p*-coumaroyl- and feruloyl-L-Tyr, using clovamide as a standard, we were able to estimate that transgenic alfalfa plants accumulating these compounds in their leaves had 250–500nmol/g FW [380±100nmol/g FW (median±MAD)] of the *trans*-*p*-coumaroyl compound and 40–90nmol/g FW [70±20nmol/g FW (median±MAD)] of the *trans*-feruloyl-compound. Thus, total hydroxycinnamoyl amide content seen in transgenic alfalfa with the highest accumulation is about an order of magnitude lower than that seen in typical mature red clover leaves.

### Transient Expression of HDT in *N. benthamiana*

**Figure 6:**
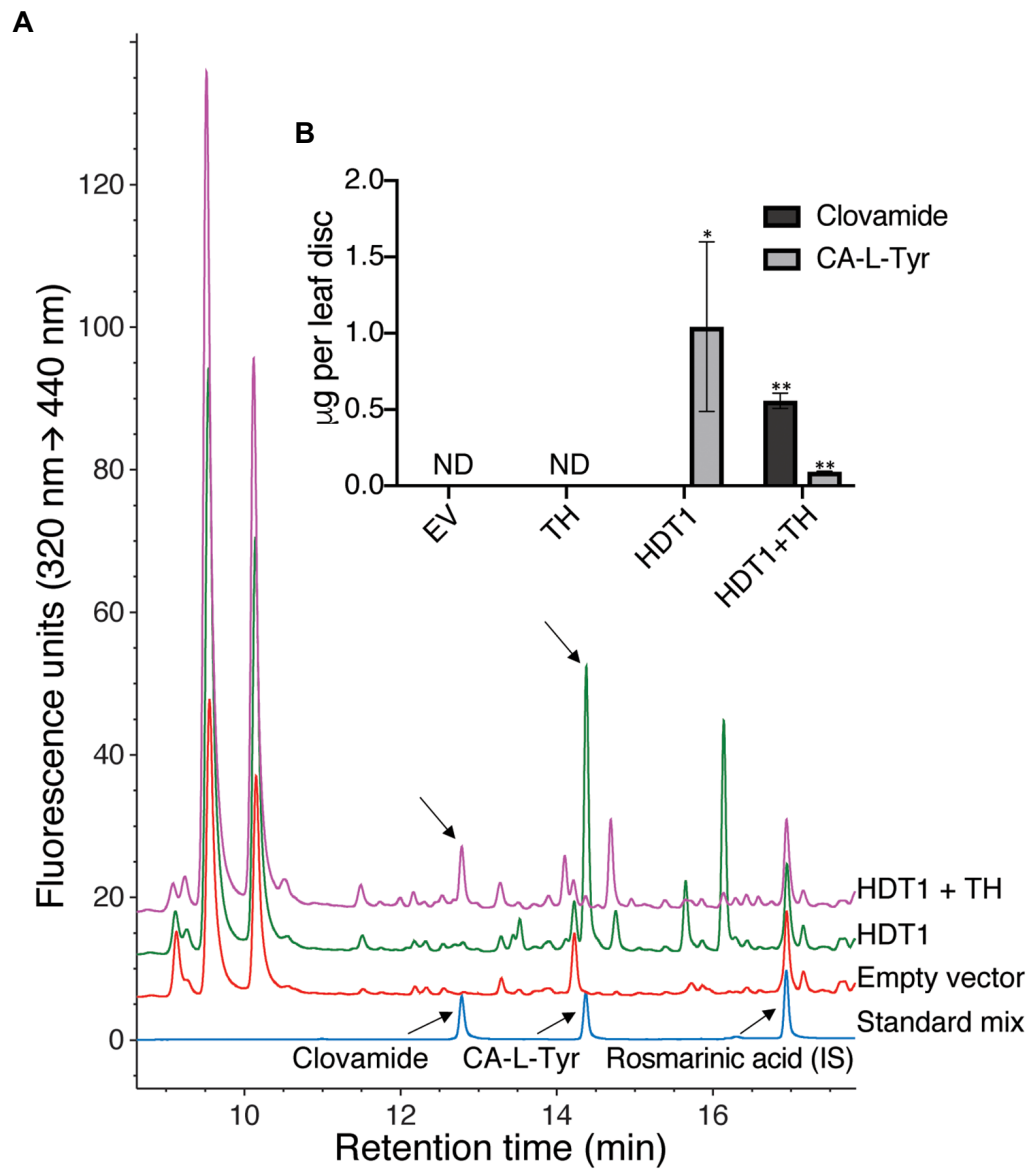
Formation of clovamide in *Nicotiana benthamiana* leaf by *A. tumefaciens*-mediated transient coexpression of HDT1 with beet tyrosine hydroxylase (TH, CYP76AD6). **(A)** HPLC-FLD chromatograms of *N. benthamiana* extracts and standards (IS, internal standard). **(B)** Clovamide and caffeoyl-L-tyrosine (CA-L-Tyr) quantification in *N. benthamiana* extracts resulting from transient expression of the empty vector control (EV), tyrosine hydroxylase (TH), HDT1, or coexpression of HDT1 and tyrosine hydroxylase (HDT1+TH). Mean±SE, *n*=4 except for HDT1+TH (*n*=3), ND=not detected. *p*<0.11 (^*^) and *p*<0.01 (^**^) using student’s t-test, in comparison with the empty vector control (EV).

To assess the effect of amino acid availability on hydroxycinnamoyl amide products formed by HDT1 in planta, HDT1 was transiently expressed in leaves of *N. benthamiana* either alone or with a tyrosine hydroxylase (“TH,” CYP76AD6) from beet capable of converting L-Tyr to L-DOPA (Polturak et al., 2016). Transient expression of HDT1 alone did not result in the formation of detectable levels of clovamide but did result in accumulation of caffeoyl-L-Tyr (Figures 6A,B). This result suggests that *N. benthamiana* leaves, like alfalfa leaves, lack sufficient endogenous L-DOPA to serve as an acyl acceptor for clovamide formation by HDT1, at least during the four-day time frame of this experiment and/or *N. benthamiana* leaves lack an endogenous hydroxylase activity toward the tyrosine moiety of caffeoyl-L-Tyr. Finally, in contrast to alfalfa, a caffeoyl derivative accumulated in the *N. benthamiana* leaves expressing HDT1, suggesting either a sufficient pool of caffeoyl-CoA for product formation by HDT1, or an endogenous hydroxylase capable of converting HDT1-produced *p*-coumaroyl *N*-amides to caffeoyl *N*-amides. When HDT1 was coexpressed with tyrosine hydroxylase from beet, clovamide did accumulate to detectable levels, indicating that the L-DOPA limitation can be overcome.

These results show that the hydroxycinnamoyl amide products formed by HDT in planta will largely depend on the available pool of both hydroxycinnamoyl-CoA donor and amino acid acceptor substrates in conjunction with the efficiency with which HDT can use the various substrates (i.e., its kinetic parameters). Furthermore, if heterologous clovamide biosynthesis is to be successfully engineered in plant species that lack sufficient hydroxylase activity toward either L-Tyr or caffeoyl-L-Tyr, coexpression of a tyrosine hydroxylase will likely be required. Such pathway engineering could be particularly useful to take advantage of clovamide’s protease inhibiting (Sullivan and Zeller, 2013), antimicrobial (Knollenberg et al., 2020), or potential health-promoting (Fallarini et al., 2009; Park et al., 2017; Tsunoda et al., 2018) properties.

## Conclusion

We identified two cDNAs from red clover (HDT1 and HDT2) that are likely responsible for accumulation of clovamide in red clover. The expression pattern of the genes in red clover corresponds with the presence of the enzyme activity, and heterologous expression in *E. coli*, alfalfa, or *N. benthamiana* produces active enzyme capable of making clovamide and related hydroxycinnamoyl amides. Although expression of the genes in alfalfa does not lead to clovamide accumulation *in vivo*, accumulation of *p*-coumaroyl- and feruloyl-L-Tyr suggests that which products accumulate *in vivo* in a particular plant species may depend in part on availability of substrates, and perhaps other modifying enzymes. The importance of substrate availability on HDT-formed products in planta is further underscored by the finding that clovamide accumulated in *N. benthamiana* leaf tissue coexpressing HDT1 and a tyrosine hydroxylase, but not in tissue expressing HDT1 only. The exact pathway(s) whereby clovamide is produced in red clover remains unclear. Given the relatively high catalytic efficiency with which L-Tyr is used, hydroxylation of *p*-coumaroyl- or caffeoyl-L-Tyr to the corresponding hydroxycinnamoyl-L-DOPA compound is an attractive model, although we have not identified such an enzyme activity. Similarly, it is conceivable that hydroxylation of *p*-coumaroyl moieties takes place at the level of the amide, although at least for HMT, it seems likely that phaselic acid is formed from caffeoyl-CoA rather than by hydroxylation of *p*-coumaroyl-malate (Sullivan and Zarnowski, 2010).

Given the relatively high degree of diversity at the primary sequence level often seen among BAHD hydroxycinnamoyl-CoA transferases, additional structural determination of members of this family of enzymes, characterized with respect to substrate specificity/kinetic parameters, would provide useful insights into the structure-function relationships of these enzymes and could allow prediction of function based on primary, 3-D, and/or modeled structures. Comparison of the structures of red clover HMT and HDT may provide especially useful information, as these two enzymes have relatively high primary sequence identity (72%) but markedly differ in substrate specificity, especially acceptor substrate. Increased understanding of the structure and function of these enzymes, and critical residues involved in substrate utilization, might also allow the creation of enzymes with new properties or improved functions.

## Data Availability Statement

The datasets presented in this study can be found in online repositories. The repository/repositories and accession number(s) can be found at https://www.ncbi.nlm.nih.gov/genbank/, MF115997, MF115998, MW605157, MW605158, and MF536893.

## Author Contributions

MS is responsible for conception and design of the study, carried out or oversaw experiments and associated data analysis with the exception of the transient expression experiment, and wrote the manuscript. BK designed, carried out, and analyzed the data for the *N. benthamiana* transient expression experiment. All authors contributed to manuscript revision, read, and approved the submitted version.

## Funding

This work was supported by the US Department of Agriculture-Agricultural Research Service (project numbers 5090-21000-055-00-D, 5090-21000-064-00-D, and 5090-21000-071-00-D) and by the Forage Genetics International *via* a Collaborative Research and Development Agreement (CRADA, 58-3K95-2-1552).

## Conflict of Interest

This work was supported in part by the Forage Genetics International. Beyond this relationship, the authors declare that the research was conducted in the absence of any commercial or financial relationships that could be construed as a potential conflict of interest.

## Publisher’s Note

All claims expressed in this article are solely those of the authors and do not necessarily represent those of their affiliated organizations, or those of the publisher, the editors and the reviewers. Any product that may be evaluated in this article, or claim that may be made by its manufacturer, is not guaranteed or endorsed by the publisher.

## Footnotes

^1^ www.ncbi.nlm.nih.gov

## Supplementary Material

The Supplementary Material for this article can be found at: https://www.frontiersin.org/articles/10.3389/fpls.2021.727461/full#supplementary-material
