# Supplementary material for: Red Clover HDT, a BAHD Hydroxycinnamoyl-Coenzyme A:L-3,4-Dihydroxyphenylalanine (L-DOPA) Hydroxycinnamoyl Transferase That Synthesizes Clovamide and Other N-Hydroxycinnamoyl-Aromatic Amino Acid Amides
Source: Front Plant Sci. 2021 Nov 9;12:727461. doi: 10.3389/fpls.2021.727461 (PMC8641662; doi:10.3389/fpls.2021.727461)
Supplement: Supplementary file 1 [file Data_Sheet_1.pdf]

```
     ms884>
   1 CAACACAGAACTTCAAGCTAGCATACCAAAAAAAAAAAAATGGTAACCATTATAGCTTCTC 60
                                           M  V  T  I  I  A  S  H

  61 ACACTGTGATTCCAGAAGAACCAACTCCACAAGGTCCATTTTGGCTCTCTGATATGGATC 120
       T  V  I  P  E  E  P  T  P  Q  G  P  F  W  L  S  D  M  D  Q

 121 AAGTGGTTCGTATCCGCGACGTACCAACTCTTTACATTTACAAAACACCAAAGAAAAACC 180
       V  V  R  I  R  D  V  P  T  L  Y  I  Y  K  T  P  K  K  N  Q
                                                               m
 181 AAGAAAACAAAAACATAGTAGAAACCTTTAAAAACTCTCTAAGCAAAATTCTTGTTCACT 240
       E  N  K  N  I  V  E  T  F  K  N  S  L  S  K  I  L  V  H  Y
     s809>
 241 ACTATCCTATAGCTGGTAGATTGTGTTACATAGAAGGTGGTAGATTAGAATTGAATCTCA 300
       Y  P  I  A  G  R  L  C  Y  I  E  G  G  R  L  E  L  N  L  N

 301 ATGCAAAAGGAGCTATTTTGGTTGAAGCTGAAACAGAAAAAACAATGAATGATTATGGTG 360
       A  K  G  A  I  L  V  E  A  E  T  E  K  T  M  N  D  Y  G  D

 361 ACTTTTCACATTTTGACACCATCAAAGAACTTGTTCCAATGATTGATTACAATCAACCAA 420
       F  S  H  F  D  T  I  K  E  L  V  P  M  I  D  Y  N  Q  P  I

 421 TTGAAGAAATTCCAAATTTTGTTGTGCAACTCACAAATTTCAAAAACAATGAAGGCTTTG 480
       E  E  I  P  N  F  V  V  Q  L  T  N  F  K  N  N  E  G  F  A
                                                        ms867>
 481 CAATTGGTGTTGCTTTTCTCCATCCTTTATCAGATGGATTGGGAGCCATTAAATTCATCA 540
       I  G  V  A  F  L  H  P  L  S  D  G  L  G  A  I  K  F  I  N

 541 ACTCATGGGCCAAAATAGCAAGAGGTGAAACACTTGAGGCTAATGAGTTACCATTTTTGG 600
       S  W  A  K  I  A  R  G  E  T  L  E  A  N  E  L  P  F  L  D
                                ms882>
 601 ATAGAAAACTTCTCAAATTTTCACACACACCTTTGGAGCCACGTTTTGAACACTTGGAGT 660
       R  K  L  L  K  F  S  H  T  P  L  E  P  R  F  E  H  L  E  L

 661 TGAAGCCACTACCACTCATTCTAGGTAGAAAAGATGCAAGTGAAGAAAAAGAGAAGAAAA 720
       K  P  L  P  L  I  L  G  R  K  D  A  S  E  E  K  E  K  K  T

 721 CTTCAGCAACATTGTTGAAACTTTCATCAGAACAAGTTGATAAGTTGAAGAAAAAAGCCA 780
       S  A  T  L  L  K  L  S  S  E  Q  V  D  K  L  K  K  K  A  N
                         <ms881
 781 ATGAAGAAGATGTTCTAGGTATCCAGAAAAAAGAGTACTCAAGGCCTTATAGTAAATTTG 840
       E  E  D  V  L  G  I  Q  K  K  E  Y  S  R  P  Y  S  K  F  E

 841 AAGTAATTAGTGCACATATATGGAGATGTGCATCTAAGGCACGTGAGCTTGAAGATAATC 900
       V  I  S  A  H  I  W  R  C  A  S  K  A  R  E  L  E  D  N  Q

 901 AAGAAAGTGTTATTAGATTCATTGCTGATGTTAAAAATAGAATGATTCCACCACTTCCTA 960
       E  S  V  I  R  F  I  A  D  V  K  N  R  M  I  P  P  L  P  K

 961 AAAACTATTTTGGGAATGCTTTGACTCAAACAGCTACTAAAGGGTATATTGGAGAAATCA 1020
       N  Y  F  G  N  A  L  T  Q  T  A  T  K  G  Y  I  G  E  I  T

1021 CATCAAAGCCTTTGGGTTACGTGGCACAAAAGATAAGGGAAGCAACTGAGTTGATAAATG 1080
       S  K  P  L  G  Y  V  A  Q  K  I  R  E  A  T  E  L  I  N  D

1081 ATGAGTATATAAGGTCACAAATTGATGTTGTTAGAAGTTTTGAACATTTGGATGATGCAC 1140
       E  Y  I  R  S  Q  I  D  V  V  R  S  F  E  H  L  D  D  A  R
```

```
1141 GAAAAATGTTTATAGGTGAAAAGGCTCGATATTTTGGTAATCCAAATTTTAATTTGACTA 1200
      K  M  F  I  G  E  K  A  R  Y  F  G  N  P  N  F  N  L  T  S
                                    <ms870        <ms815
1201 GTTGGTTAAGTATGCCTGTTTATGAAGCTGATTTTGGATGGGGAAACCTAATTACTTTG 1260
      W  L  S  M  P  V  Y  E  A  D  F  G  W  G  K  P  N  Y  F  G

1261 GATTAGCTGATGTCTCACCACATGATAGAGCAGTCATTCTTCTTAGTCCTGATGATGATG 1320
      L  A  D  V  S  P  H  D  R  A  V  I  L  L  S  P  D  D  D  G

1321 GATCTGTTCTTGTGTCTTTCCATTTTCAGATTGCACATATGGAGCTTTTCAACAAGTATT 1380
      S  V  L  V  S  F  H  F  Q  I  A  H  M  E  L  F  N  K  Y  F

1381 TTTATGAGGAGATATGAAATAGGGGTGGTTTTTGGGTCAATTTTTGACCCAAAATCACCC 1440
      Y  E  E  I  *
          <ms885
1441 TCTAAGTTGGT 1451
```

Figure S1. Nucleotide and predicted amino acid sequence of HDT1 cDNA (Genbank MF115997). Oligonucleotide primer annealing sites are underlined and the corresponding primer names (see Table 1) are indicated above the nucleotide sequence at the 5' primer end. Forward primers (annealing to the antisense strand) are indicated by “>” and reverse primers (annealing to the sense strand) are indicated by “<”.

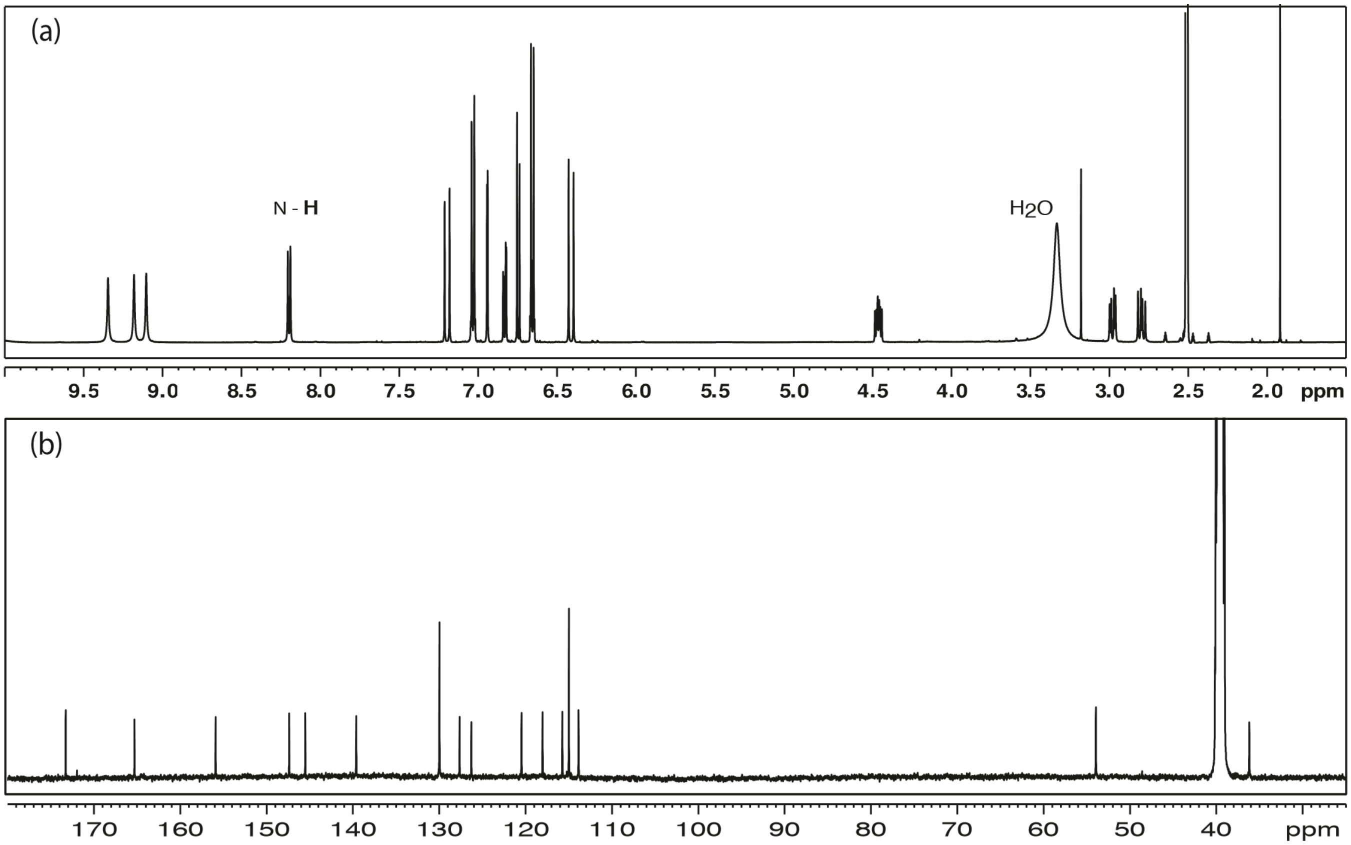

Figure S2. NMR evaluation of caffeoyl-L-Tyr generated by HDT1 in vitro. Caffeoyl-L-Tyr was synthesized in a 6 mL reaction by incubating 1 mM caffeoyl-CoA, 3 mM L-tyrosine, and HDT1 (25 µg/mL expressed and purified from E. coli as described in the Materials and methods section) in a buffer of 100 mM sodium phosphate and 25 mM ascorbic acid, pH 8.0. Following a 90 min incubation at 30°C, the reaction was acidified by the addition of 1/10th volume 1 N HCl. The acidified reaction mixture was applied to a 100 mg ENVI-18 solid phase extraction column (Supelco, Bellafonte, PA, USA) preequilibrated with 3 X 1 mL methanol and 3 X 1 mL 1% (v/v) acetic acid in water, the column was washed with 3 X 1 mL 1% (v/v) acetic acid in water and 3 X 1 mL 0.1% (v/v) acetic acid in water, and product was eluted with 1 mL ethanol. The eluate was dried under a stream of nitrogen and the residue dissolved in DMSO-d_6. NMR spectra were recorded on a 500MHz Bruker Biospin Avance III HD spectrometer (Bruker, Billerica, MA, USA) equipped with a 5 mm inverse gradient TCI cryoprobe at 298 K. ^{1}H NMR (500 MHz, DMSO-d_6) (a) and ^{13}C NMR (125 MHz, DMSO d_6) (b) spectra of the caffeoyl-L-Tyr product are shown.

Below, chemical shifts (δ, ppm) for the in vitro product are referenced to the residual undeuterated solvent peak, 2.5 for ^{1}H NMR and 39.5 for ^{13}C NMR. The following abbreviations are used to denote the multiplicities: s = singlet, d = doublet, t = triplet, and m = multiplet, and are followed by their coupling constants (*J*, Hz).

^{1}H NMR (500 MHz, DMSO-d_6): δ 2.79 (1H, dd, 9.5, 14.0), 2.98 (1H, dd, 4.5, 13.5), 4.44-4.48 [1H, m), 6.41 (1H, d, 15.8), 6.66 (2H, d, 8.6), 6.75 (1H, d, 8.1), 6.83 (1H, dd, 2.1, 8.3), 6.94 (1H, d, 2.1), 7.02 (2, 2H, 8.5), 7.20 (1H, d, 15.7), 8.2 (1H, d, H-N), 9.10, 9.18, 9.35 (3 × 1H, 3 s),

12.61 (1H, s); ^{13}C NMR (125 MHz, DMSO-d_6): δ 36.1, 53.9, 113.8, 115.0, 115.7, 118.0, 120.4, 126.3, 127.6, 130.0, 139.6, 145.5, 147.4, 155.9, 165.3, 173.3.

Spectroscopic data is consistent with that reported for (-)-N-[3′,4′-Dihydroxy-(E)-cinnamoyl]-L-tyrosine in the literature (Stark and Hofmann, 2005).

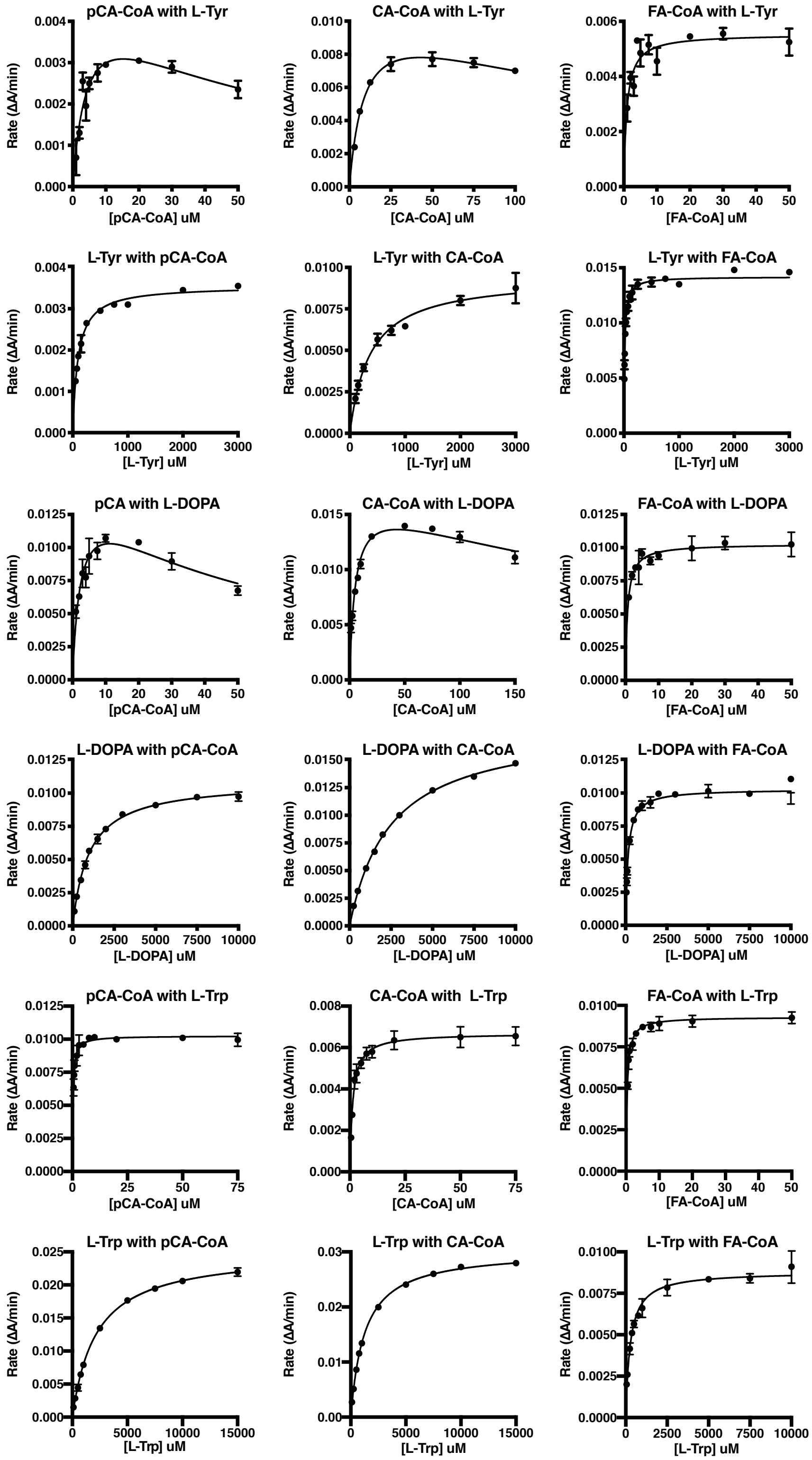

Figure S3. Kinetic curves showing rate (as ΔA_{412nm}/min) versust substrate concentration. Donor and acceptor substrates are as indicated. ΔA_{412nm}/min values were converted to katal as described in Sullivan and Bonawitz, 2018 for determination calculation of $k_{cat.}$
